# Perovskite Thin‐Film Transistors for Ultra‐Low‐Voltage Neuromorphic Visions

**DOI:** 10.1002/advs.202410015

**Published:** 2024-11-06

**Authors:** Yang Rong, De Yu, Xin Zhang, Tao Wang, Jie Wang, Yuheng Li, Tongpeng Zhao, Ruiqin He, Yuxin Gao, Can Huang, Shumin Xiao, Jingkai Qin, Sai Bai, Huihui Zhu, Ao Liu, Yimu Chen, Qinghai Song

**Affiliations:** ^1^ Ministry of Industry and Information Technology Key Lab of Micro‐Nano Optoelectronic Information System Guangdong Provincial Key Laboratory of Semiconductor Optoelectronic Materials and Intelligent Photonic Systems Harbin Institute of Technology (Shenzhen) Shenzhen Guangdong 518055 China; ^2^ Institute of Fundamental and Frontier Sciences University of Electronic Science and Technology of China Chengdu Sichuan 611731 China; ^3^ Sustainable Energy and Environment Thrust The Hong Kong University of Science and Technology (Guangzhou) Guangzhou Guangdong 511400 China; ^4^ School of Integrated Circuits Harbin Institute of Technology (Shenzhen) Shenzhen Guangdong 518055 China; ^5^ School of Physics University of Electronic Science and Technology of China Chengdu Sichuan 611731 China

**Keywords:** artificial synapses, interfaces engineering, low operating voltage, neuromorphic visions, perovskite thin‐film transistors

## Abstract

Perovskite thin‐film transistors (TFTs) simultaneously possessing exceptional carrier transport capabilities, nonvolatile memory effects, and photosensitivity have recently attracted attention in fields of both complementary circuits and neuromorphic computing. Despite continuous performance improvements through additive and composition engineering of the channel materials, the equally crucial dielectric/channel interfaces of perovskite TFTs have remained underexplored. Here, it is demonstrated that engineering the dielectric/channel interface in 2D tin perovskite TFTs not only enhances the performance and operational stability for their utilization in complementary circuits but also enables efficient synaptic behaviors (optical information sensing and storage) under an extremely low operating voltage of −1 mV at the same time. The interface‐engineered TFT arrays operating at −1 mV are then demonstrated as the preprocessing hardware for neuromorphic visions with pattern recognition accuracy of 92.2% and long‐term memory capability. Such a low operating voltage provides operational feasibility to the design of large‐scale‐integrated and wearable/implantable neuromorphic hardware.

## Graphical abstract

Engineering the dielectric/channel interface in 2D tin perovskite thin‐film transistors (TFTs) not only enhances the performance and operational stability for their utilization in complementary circuits but also enables efficient synaptic behaviors under −1 mV. The interface‐engineered TFT arrays are demonstrated as the preprocessing hardware for neuromorphic visions with pattern recognition accuracy of 92.2% and long‐term memory capability.

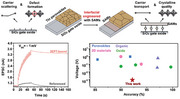

## 1 Introduction

Thin‐film transistors (TFTs) using perovskites as the channel materials exhibit outstanding carrier transport characteristics for their applications in complementary circuits.^[^ ^1^ ^]^ Meanwhile, nonvolatile memory effects and photosensitivity can be simultaneously integrated into the high‐performance perovskite TFTs due to the intrinsic properties of perovskite materials, attracting attention in the field of neuromorphic computing.^[^ ^2^ ^]^ Given the low‐temperature solution processability, facile scalability, hetero‐integration ability, and mechanical flexibility of perovskites, multifunctional TFTs based on perovskites can potentially be used as high‐performance hardware with high integration density for the above two fields. While substantial efforts have been made to enhance the device performance by manipulating perovskite channels with additive and composition engineering, the properties of the dielectric/channel interface are rarely studied. In particular, defects at the dielectric/channel interface exert a significant influence on the carrier transport in TFTs, which thereby determines the performance and functionalities of TFTs.

**Figure 1:**
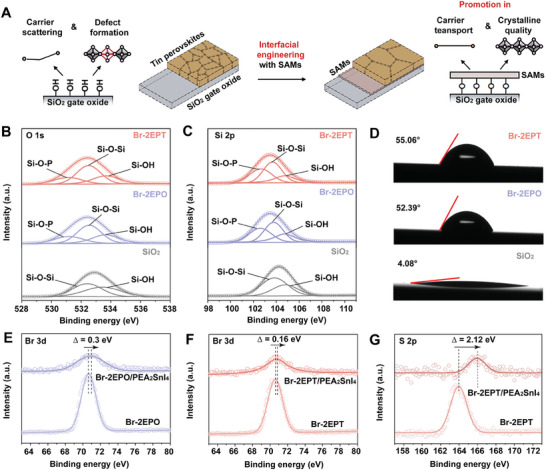
Characterizations of SiO_2_ gate dielectric with interfacial engineering. A) Schematic illustration of the proposed interfacial engineering of tin perovskite TFTs by SAMs. B) O 1*s* and C) Si 2*p* core level spectra of the UV‐treated, Br‐2EPO‐coated, and Br‐2EPT‐coated SiO_2_ substrates. D) Contact angle measurements of the UVO‐treated, Br‐2EPO‐coated, and Br‐2EPT‐coated SiO_2_ substrates. Br 3*d* core level spectra of E) Br‐2EPO, F) Br‐2EPT, and their corresponding mixture with PEA_2_SnI_4_. G) S 2*p* core level spectra of Br‐2EPT and Br‐2EPT/PEA_2_SnI_4_ mixture.

For perovskite TFTs, SiO_2_ is predominately used as the gate dielectric due to its high reliability and integration compatibility. However, hydrophilic treatments of the gate oxide are usually required before the deposition of perovskites to ensure conformal coating, thereby generating excess hydroxyl groups (silanols) at the surface of SiO_2_ (**Figure** **1**A). On the one hand, carriers can be scattered and captured by silanols that serve as charged centers at the interface, which is commonly responsible for the sub‐optimal performance of transistors such as mobility and operational stability.^[^ ^3^ ^]^ On the other hand, buried interfaces of perovskites are sensitive to underlying substrates that serve as templates for crystallization and, therefore, excess silanols induce additional defects in the sequentially deposited perovskite channel,^[^ ^4^ ^]^ which further complicate the dielectric/channel interface.

In this study, interfacial engineering with synergistic passivation effects by using self‐assembly monolayers (SAMs) is proposed to address the dielectric/channel interface issues in 2D tin perovskite phenethylammonium tin iodide (PEA_2_SnI_4_) TFTs. Specifically, excess hydroxyl groups (silanols) on the SiO_2_ gate dieletrics can be effectively passivated by the phosphonic acid moiety in the anchoring groups of SAMs while terminal groups of SAMs can further promote the crystalline quality of tin perovskites by Lewis acid‐base interactions. Therefore, defects on the surface of the SiO_2_ gate dielectric and in the tin perovskite channel are simultaneously suppressed to mitigate carrier scattering and recombination at the dielectric/channel interface which promotes carrier transport and inhibits the decomposition of materials. As a result of the interfacial engineering, device performance of tin perovskite TFTs, including carrier mobility, on/off current ratio, threshold voltage, dual‐scan hysteresis, subthreshold swing, and operational stability, are effectively optimized.

More importantly, the interface‐engineered TFTs are also found to maintain efficient carrier modulation and transport under extreme electrical operation conditions where source‐drain voltages (*V_DS_*) can be reduced to as low as −1 mV, suggesting that they can work as low‐voltage synaptic TFTs. Considering the large‐scale integration of synaptic hardware for neuromorphic computing, the operating voltage of a single artificial synapse should be minimized for operation feasibility and power consumption. However, carrier trapping/detrapping processes enabling the synaptic behaviors in perovskites are vulnerable to the conditions of dielectric/channel interface under low operating voltages since carriers can easily be scattered and recombined under weak electric fields. Benefiting from the efficient carrier transport under low *V_DS_*, the resulting TFTs can work as photonic synapses under an operating voltage (−1 mV) that is even lower than the excitatory postsynaptic potential to activate biological synapses (>15 mV).^[^ ^5^ ^]^ Consequently, a neuromorphic visual system based on interface‐engineered tin perovskite synaptic TFT arrays operating under −1 mV and artificial neural networks (ANNs) is demonstrated for pattern learning, memory, and recognition with recognition accuracy reaching 92%. Such a low operating voltage of the synaptic TFT arrays is 1 to 3 magnitudes lower than those of synaptic hardware based on conventional materials, 2D materials, organics, and perovskites.

## 2 Results and Discussion

### 2.1 Interfacial Engineering Mechanisms

To synergistically passivate the excess silanols at the surface of SiO_2_ and promote the sequential growth of the stacked tin perovskite layer, interfacial engineering with SAMs is proposed (Figure 1A). Specifically, two types of SAMs with phosphonic acid anchoring groups, namely Br‐2EPO with O‐donor terminals and Br‐2EPT with S‐donor terminals (Figure S1, Supporting Information), are adopted. On the one hand, phosphonic acid anchoring groups can form covalent bonds with silanols through the silanization process, thereby effectively reducing the amount of silanols. X‐ray photoelectron spectroscopy (XPS) measurements are conducted on UVO‐treated, Br‐2EPO‐coated, and Br‐2EPT‐coated SiO_2_ substrates to reveal the passivation of silanols. O 1*s* core level peak (Figure 1B) of the UVO‐treated SiO_2_ substrate can be deconvoluted into two subpeaks corresponding to silicon atoms in SiO_2_ lattices (Si‐O‐Si) and silanols (Si‐O‐H)^[^ ^6^ ^]^ and the ratios of Si‐O‐Si and Si‐O‐H are calculated to be 51.8% and 48.2% (Table S1, Supporting Information), respectively, indicating the presence of a high density of silanols on the surface. Upon the deposition of SAMs, a third subpeak (Si‐O‐P)^[^ ^7^ ^]^ emerged due to the silanization process between silanols and phosphonic acid anchoring groups in SAMs,^[^ ^8^ ^]^ and the ratios of Si‐O‐H in both types of SAMs‐coated SiO_2_ substrates significantly decrease to ≈27% (Table S1, Supporting Information), demonstrating the passivation of silanols by SAMs molecules. Si 2*p* core level peaks of the three types of SiO_2_ substrates exhibit a similar evolutionary trend (Figure 1C; and Table S2, Supporting Information), further identifying the passivation of silanols. Besides, contact angles of Br‐2EPO‐coated and Br‐2EPT‐coated SiO_2_ substrates significantly increase from 4.08° of UV‐treated SiO_2_ substrate to 52.39° and 55.06° (Figure 1D), respectively, which is in agreement with the passivation of silanols by SAMs characterized by XPS. Therefore, we speculate that carrier scattering by silanols and silanol‐induced defects in the ensuing perovskite TFTs would be potentially suppressed.

On the other hand, O‐donor and S‐donor terminals in SAMs can serve as Lewis bases^[^ ^9^ ^]^ for the coordination with tin perovskites so that the formation of tin vacancies (Sn^4+^) can be suppressed.^[^ ^10^ ^]^ Meanwhile, Br atoms in both types of SAMs can also interact with tin perovskites through the formation of hydrogen bondings, further contributing to the passivation of defects.^[^ ^11^ ^]^ In light of the possible interactions between SAMs and tin perovskites, Br 3*d* and S 2*p* core level spectra of Br‐2EPO and Br‐2EPT, as well as their corresponding mixture with PEA_2_SnI_4_, are studied. Considering the possible oxygen contamination in samples, analysis of O 1*s* core level spectrum is discarded. Both Br 3*d* peaks in Br‐2EPO/PEA_2_SnI_4_ and Br‐2EPT/PEA_2_SnI_4_ (Figure 1E,F), as well as the S 2*p* peak in Br‐2EPT/PEA_2_SnI_4_ (Figure 1G), exhibit obvious shifts when compare with those in SAMs, demonstrating the interactions between SAMs and tin perovskites. Meanwhile, the shifts of Br 3*d* peaks are relatively less prominent, which is in accordance with the formation of weak hydrogen bondings. The above interactions between SAMs and perovskites are expected to further modulate the crystalline quality of channel perovskite materials deposited on top, which would additionally contribute to the enhancement of carrier transport and device performance.

**Figure 2:**
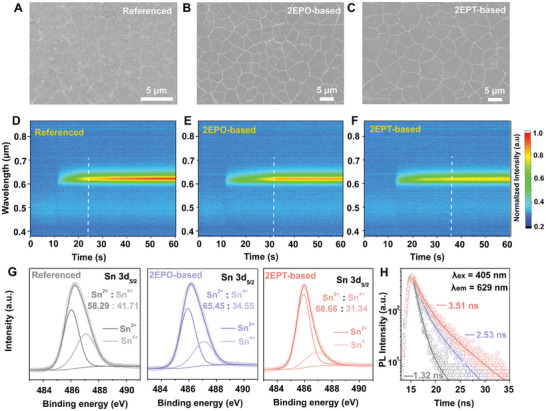
Characterizations of PEA_2_SnI_4_ channel with interfacial engineering. SEM images of the A) referenced, B) 2EPO‐based, and C) 2EPT‐based samples. In situ PL mapping of the D) referenced, E) 2EPO‐based, and F) 2EPT‐based samples. G) Sn 3*d*_5/2_ core level spectra and H) TRPL spectra of the referenced, 2EPO‐based, and 2EPT‐based samples.

Given the passivation of excess silanols on SiO_2_ and the interactions between SAMs and tin perovskites, the crystallization of channel material PEA_2_SnI_4_ can be significantly affected. Scanning electron microscopy (SEM) image of the PEA_2_SnI_4_ deposited on UVO‐treated SiO_2_ substrate (denoted as the referenced sample) exhibited non‐uniform surface morphology and high density of grooves and pinholes (**Figure** **2**A). A distinct enhancement of film uniformity and grain compactness can be identified in PEA_2_SnI_4_ deposited on Br‐2EPO‐coated (Figure 2B) and Br‐2EPT‐coated (Figure 2C) SiO_2_ substrates (denoted as 2EPO‐based and 2EPT‐based sample, respectively). At the same time, the roughness of PEA_2_SnI_4_ significantly reduced from 21.4 nm of the referenced sample to 12.7 nm of the 2EPO‐based sample and 11.9 nm of the 2EPT‐based sample (Figure S2, Supporting Information).

To understand the enhancement in the morphology of SAMs‐based PEA_2_SnI_4_, in situ photoluminescence (PL) measurements are carried out to study to crystallization of PEA_2_SnI_4_ thin films (Figure 2D–F). For the referenced sample, the intensity of the PL peak corresponding to the perovskite phase commences to saturate at the 24′ s after the onset of spinning, indicating a rapid nucleation and crystallization of PEA2SnI4 due to the presence of excess silanols that result in a high‐density of nuclei on the surface of SiO2. In contrast, in situ PL spectra of both 2EPO‐based and 2EPT‐based PEA2SnI4 thin films suggest that the formation speed of the perovskite phase is effectively retarded, with crystallization commencing at the 31′ and 36′ s after the onset of spinning. The retarded nucleation and crystallization processes agree well with the enhancement of thin‐film morphology, which can be partially attributed to the passivation of silanols. Besides, SAMs are also anticipated to promote the crystallization of PEA_2_SnI_4_ through the interactions between terminals of SAMs and tin perovskites. Specifically, the S‐donor in Br‐2EPT is considered a stronger Lewis base than the O‐donor in Br‐2EPO,^[^ ^13^ ^]^ thereby possessing an enhanced perovskite affinity, explaining the further promotion in grain size (Figure 2C), roughness (Figure S2C, Supporting Information), and crystallization rate (Figure 2F) of 2EPT‐based sample when compared with those of 2EPO‐based sample. Consequently, PEA_2_SnI_4_ thin films that serve as the channel in TFTs exhibit improved crystalline quality. Meanwhile, Sn 3*d*_5/2_ core level spectra composed of Sn^2+^ (≈485.9 eV) and Sn^4+^ (tin vacancy, ≈486.9 eV) subpeaks are characterized by XPS,^[^ ^14^ ^]^ and the contents of Sn^4+^ in PEA_2_SnI_4_ thin films based on different types of gate substrates are evaluated. The content of Sn^4+^ decreases from 41.7% in the referenced sample to 34.5% in the 2EPO‐based sample and 31.3% in the 2PET‐based sample (Figure 2G), suggesting the suppression of tin vacancies that act as the main source of deep‐level traps in tin perovskites^[^ ^15^ ^]^ due to the interfacial engineering by SAMs. Meanwhile, the improvement of crystalline quality is also supported by the enhancement of intensity and the reduction of linewidth of the diffraction peaks in 2EPO‐based and 2EPT‐based samples (Figure S3, Supporting Information).

Carrier transport in the channels directly determines the performance, as well as the functionality, of TFTs, and it is strongly affected by defects in dielectric/channel interface.^[^ ^16^ ^]^ As a result of the proposed interfacial engineering, carrier dynamics of channel material PEA_2_SnI_4_ that reflect the carrier transport processes can be effectively promoted. Specifically, time‐resolved photoluminescence spectroscopy measurements (Figure 2H) show that carrier lifetimes of 2EPO‐based (2.53 ns) and 2EPT‐based (3.51 ns) PEA_2_SnI_4_ thin films are distinctly higher than that of the referenced sample (1.32 ns) (see details in the Table S3, Supporting Information). Similar trends are also found in the comparisons of intensity of steady‐state photoluminescence peaks (Figure S4A, Supporting Information) and photoluminescence quantum yields (Figure S4B, Supporting Information). The above enhancements in the carrier dynamics suggest the reduction of non‐radiative carrier losses, which can be attributed to the suppression of defects in dielectric/channel interface.

### 2.2 Carrier Transport Promotions by Interfacial Engineering in PEA_2_SnI_4_ TFTs

**Figure 3:**
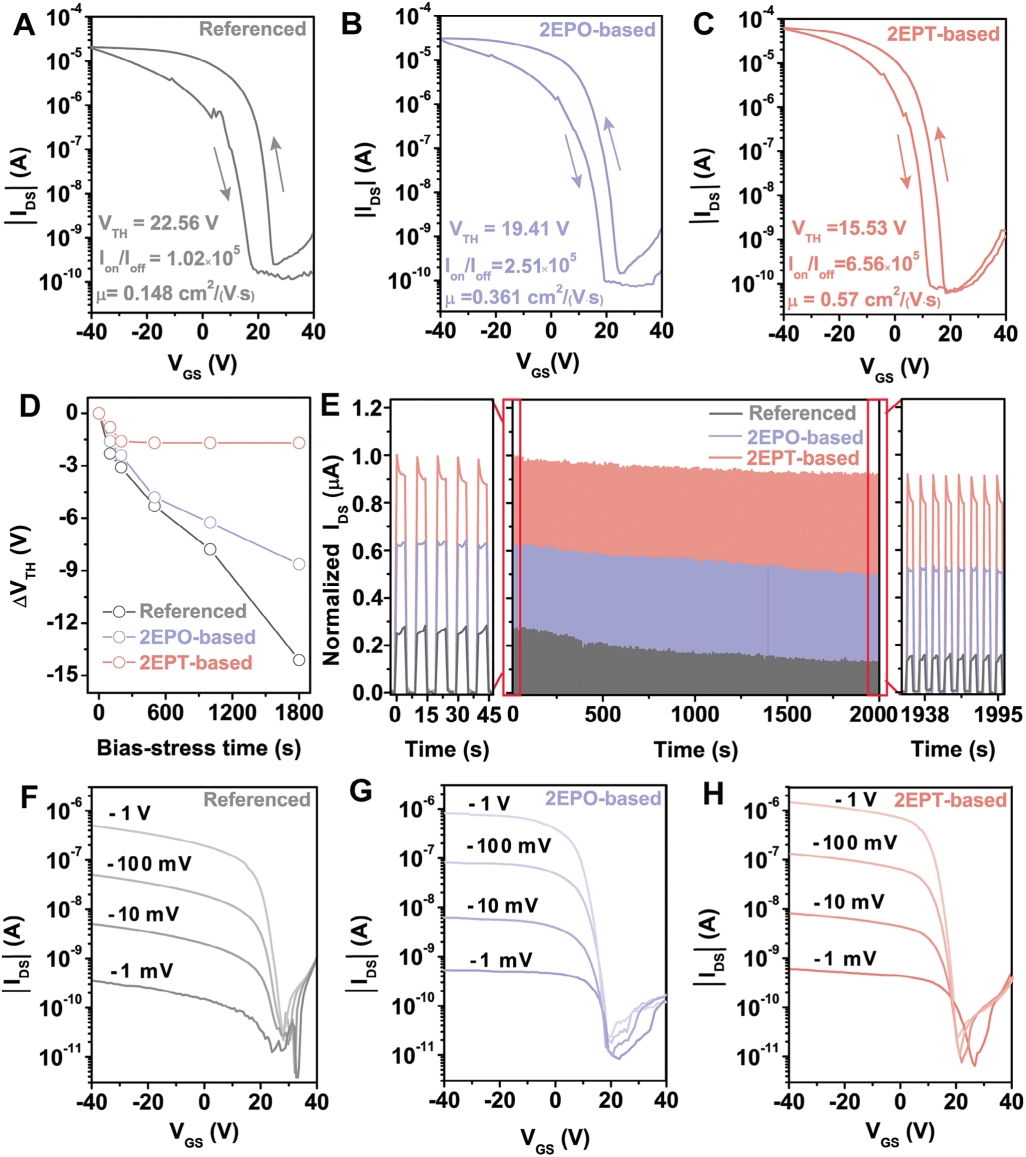
Characterizations of PEA_2_SnI_4_ TFTs with interfacial engineering. Typical transfer curves of TFTs based on the A) referenced, B) 2EPO‐based, and C) 2EPT‐based devices (*V_DS_* = –40 V). D) Variations of *V_TH_* of the referenced, 2EPO‐based, and 2EPT‐based devices under constant bias stress (*V_GS_* = –40 V, *V_DS_* = –40 V). E) Continuous on/off switching test of one representative referenced, 2EPO‐based, and 2EPT‐based devices (*V_GS_* = –40 V, *V_DS_* = –40 V). Transfer curves of F) referenced, G) 2EPO‐based, and H) 2EPT‐based devices under low *V_DS_* conditions (–1 V to –1 mV).

Different types of PEA_2_SnI_4_ TFTs are then fabricated in a bottom gate/bottom contact structure (Figure S5, Supporting Information) and their performance parameters are characterized and summarized in Table S4 (Supporting Information). Transfer curves of the referenced (**Figure** **3**A), 2EPO‐based (Figure 3B), and 2EPT‐based (Figure 3C) PEA_2_SnI_4_ TFTs display a standard p‐type character. The off‐state currents of 2EPO‐based and 2EPT‐based devices are both lower than that of the referenced device while the 2EPT‐based device shows the lowest value, which is in agreement with the reduction of Sn^4+^ in channels (Figure 2G).^[^ ^1d^ ^]^ Due to the same reason, threshold voltages (*V_TH_*) of 2EPO‐based (19.41 V) and 2EPT‐based (15.53 V) devices are also reduced when compared with that of the referenced device (22.56 V) (see details in the Figure S6, Supporting Information), which is favorable for reducing the energy consumption during device operations. Benefiting from the enhanced carrier transport at the dielectric/channel interface, the on‐state currents of SAMs‐based devices are higher than that of the referenced device. Consequently, the on/off current ratios of the 2EPT‐based device (6.56 × 10^5^) and 2EPO‐based (2.51 × 10^5^) are promoted when compared with that of the referenced (1.02 × 10^5^) devices. As a result of the enhanced carrier transport character by interfacial engineering, carrier mobilities (*μ*) extracted from the transfer curves show that the 2EPT‐based device (0.57 cm^2^ V^−1^ s^−1^) outperforms the other two types of devices while the carrier mobility of 2EPO‐based device (0.361 cm^2^ V^−1^ s^−1^) is also superior to that of the referenced device (0.148 cm^2^ V^−1^ s^−1^).

At the same time, dual‐scan hysteresis resulting from the migration of defects (in the form of ions)^[^ ^17^ ^]^ is also suppressed in SAMs‐based devices, which can be ascribed to the passivation of defects in the PEA_2_SnI_4_ channel through interfacial engineering. Based on the voltage difference (*∆V*) at source‐drain current (*I*_DS_) of 10^−7^ A extracted from the dual‐scan hysteresis (referenced: 11.6 V, 2EPO‐based: 7.2 V, and 2EPT‐based: 5.6 V), the number of trapped carriers (*∆N*) of the referenced, 2EPO‐based, and 2EPT‐based devices are respectively calculated to be 2.11 × 10^12^, 1.31 × 10^12^, and 1.02 × 10^12^ cm^−2^, further demonstrating the reduction of traps by interfacial engineering. Subthreshold swing (*SS*) that determines the operational speed of transistors is also strongly affected by defects at the dielectric/channel interface through carrier scattering and capture. A reduction of average *SS* values can be extracted following the trend in the referenced (2.60 V dec^−1^), 2EPO‐based (2.27 V dec^−1^), and 2EPT‐based devices (1.46 V dec^−1^) (Figure S7A, Supporting Information). Furthermore, average trap density (*N_t_*) calculated using the extracted average *SS* values of the referenced, 2EPO‐based, and 2EPT‐based devices are then respectively shown to be 7.73 × 10^12^, 6.72 × 10^12^, and 4.27 × 10^12^ cm^−2^ eV^−1^ (Figure S7B, Supporting Information), which is in agreement with *∆N* extracted from the *∆V*. Enhancement of output current and linearity from the output curves can also be identified in 2EPO‐based and 2EPT‐based devices when compared with those of the referenced device, agreeing with the promotion of carrier transport observed from the transfer curves (Figure S8, Supporting Information). Utilizing the optoelectronic properties of tin perovskites, phototransistors based on our TFTs are demonstrated (Figure S9, Supporting Information) and interfacial engineering is shown to enhance the performance of phototransistors, including photosensitivity, responsivity, and specific detectivity (Figure S10, Supporting Information). Meanwhile, 2EPT‐based devices outperform 2EPO‐based devices as well.

Besides, interfacial engineering also improves the operational stability of tin perovskite TFTs. Defects in both dielectric and channel at the interface are usually considered one of the main reasons for performance degradation during operations.^[^ ^3c^ ^]^ Long‐term bias stress measurements of the three types of devices (Figure 3D; Figure S11, Supporting Information) under a constant negative gate and drain voltages (*V_GS_* = –40 V, *V_DS_* = –40 V) are carried out in a N_2_ filled glovebox and the referenced device experience a substantial negative *V_TH_* shift (14 V) after 1800 s bias stress test. Comparably, both 2EPO‐based and 2EPT‐based devices exhibit a suppressed *V_TH_* shift, while a *V_TH_* shift less than 1.5 V after continuous bias for over 1800 s can be identified in the 2EPT‐based device. Meanwhile, dynamic on/off switching measurements show that the ratios of *I_DS_* reduction after 2000 cycles in the referenced, 2EPO‐based and 2EPT‐based devices are determined to be 53.5%, 19.4%, and 8%, respectively (Figure 3E). The above results clearly show that passivating defects in both dielectric and channel through interfacial engineering improve the operational stability of tin perovskite TFTs. In addition to the operational stability, the environmental stability of devices with interfacial engineering is effectively enhanced as well (Figure S12, Supporting Information).

More importantly, transfer curves of the three types of devices with different *V_DS_* ranging from –40 V to –1 mV are characterized. All three types of devices exhibit decent carrier transport characters under high *V_DS_* ranging from –40 to –1 V (Figure S13, Supporting Information) since the carrier transport processes are mainly controlled by the relatively strong electric field between the drain and the source. When *V_DS_* further reduces to lower than –1 V, especially at –1 mV, the carrier transport capability of the referenced device is impaired, as evident by the weakened modulation of *I*_DS_ with the change of *V*_GS_ (Figure 3F). We anticipate that carriers under such a weak electric field in the referenced device are susceptible to the presence of excess defects in both dielectric and channel at the interface through scattering and recombination. However, transfer curves of both 2EPO‐based and 2EPT‐based devices can maintain an effective modulation of *I*_DS_ with the change of *V*_GS_ even under *V*_DS_ of –1 mV (Figure 3G,H), indicating that the carrier transport at the dielectric/channel interface is less affected by defects at the interface due to the effective defect passivation by interfacial engineering. Such a promoted carrier modulation chain TFTs under low *V*_DS_ in TFTs is highly desirable for their applications.^[^ ^1b^ ^]^

### 2.3 Interfacial Engineering of PEA_2_SnI_4_ TFTs for Low‐Voltage Neuromorphic Hardware

2D perovskites exhibit intrinsic nonvolatile memory effects due to the carrier trapping/detrapping processes resulting from their internal quantum‐well structure,^[^ ^18^ ^]^ which are favorable for constructing photonic synapses for neuromorphic visions. However, carrier trapping/detrapping processes are vulnerable to the presence of defects, especially under low operating voltages and weak electric fields.^[^ ^19^ ^]^ Considering the efficient carrier transport under low *V_DS_* of –1 mV, interface‐engineered PEA_2_SnI_4_ TFTs can potentially exhibit efficient synaptic behaviors under the same level of operating voltage, which is favorable to the operational feasibility and energy consumption of photonic synapses toward applications with large‐scale integration.

Before that, synaptic behaviors under a high operating voltage (*V_DS_* of –20 V) are first characterized. All three types of devices exhibit typical short‐term potentiation (STP) with incident light stimulation, including excitatory postsynaptic current (EPSC) under a single light pulse (Figure S14A, Supporting Information) and paired‐pulse facilitation (PPF) with two consecutive light pulses (Figure S14B,C, Supporting Information). SAMs‐based devices possess higher EPSC magnitudes and PPF index than those of the referenced device, and the 2EPT‐based device also outperforms the 2EPO‐based device, which is in agreement with the promotions in material properties (Figure 2) and TFT performances (Figure 3) by interfacial engineering. Increment of EPSC magnitude can be achieved by optical modulations (Figure S15, Supporting Information), including incident light pulse duration, number, and frequency, to mimic the enhanced learning processes in the human brain. Meanwhile, EPSC decays are retarded with the increment of EPSC magnitude, representing the transition from STP to long‐term potentiation (LTP) that mimics the memory process in brains. Such LTP can even last for more than an hour with consecutive light stimulation (Figure S16, Supporting Information). EPSC decays in both 2EPO‐based and 2EPT‐based devices are longer than that of the referenced device under the same stimulation conditions (Figure S17, Supporting Information), indicating the enhancement of memory, as well as the reduction of learning cost, due to the suppression of carrier losses during carrier detrapping processes. Except for optical modulations, EPSC magnitude can be further controlled by electrical modulation of *V_GS_* (Figure S18, Supporting Information), and optical‐potentiation‐electrical‐depression operations (Figure S19, Supporting Information) and learning‐experience behavior (Figure S20, Supporting Information) can also be realized. The above results suggest that PEA_2_SnI_4_ TFTs can serve as photonic synapses under sufficiently high operating voltages that drive the carrier detrapping processes, and interfacial engineering can enhance their performance by suppressing carrier scattering and non‐radiative losses.

**Figure 4:**
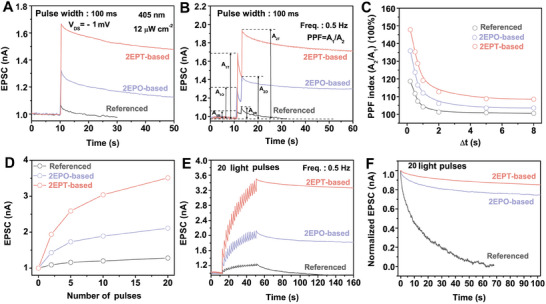
Synaptic behaviors of PEA_2_SnI_4_ TFTs under *V_DS_* of −1 mV. A) EPSC and B) PPF behaviors of the three types of devices triggered by a single light pulse and a pair of light pulses, respectively. A_1_ and A_2_ represent the amplitude of the first and second EPSC, respectively. C) Pulse‐interval‐dependent PPF index (A_2_/A_1_) of the three types of devices. D) Pulse‐number‐dependent EPSC of the three types of devices. E) EPSC behaviors and F) the corresponding EPSC decays of the three types of devices triggered by 20 consecutive light pulses with a frequency of 0.5 Hz. Wavelength, pulse width, and light intensity of light pulses in all characterization are 405 nm, 100 ms, and 12 µW cm^−2^, respectively.

More importantly, synaptic behaviors of the three types of devices under an operating voltage of −1 mV are then characterized. With a single light pulse, EPSC is extremely weak in the referenced device (**Figure** **4**A), which is likely due to the severe carrier scattering and non‐radiative recombination at the defective dielectric/channel interface. Contrarily, EPSC signals, as well as EPSC decay, can be seen in SAMs‐based devices while the EPSC signal of the 2EPT‐based device is also stronger than that of the 2EPO‐based device (Figure 4A), indicating that carriers in SAMs‐based devices are less likely to be dominated by scattering and non‐radiative recombination even under extremely weak electric fields. Reinforced learning behaviors of the synaptic TFTs are further studied with PPF. Compared with the referenced device, SAMs‐based devices exhibit prominent EPSC enhancement when subjected to paired light stimulation (Figure 4B), and the corresponding PPF index of the referenced, 2EPO‐based, and 2EPT‐based devices are respectively calculated to be 118.7%, 135.9%, and 147.8% (Figure 4C). By extending the time interval between two consecutive pulses, PPF index of the referenced device swiftly decay to ≈100% in the referenced device (Figure 4C), demonstrating that the second light pulse can hardly contribute to the EPSC increment. However, the referenced device under *V_DS_* of −20 V retains a PPF index of ≈105% even when the interval between paired pulses is set to 20 s (Figure S14C, Supporting Information). Such a difference between EPSC under different operating voltages suggests that the learning process in the referenced device under low operating voltage is severely restricted.

EPSC increment also quickly saturates in the referenced device by increasing the number of light pulses (Figure 4D; Figure S21, Supporting Information), which is in agreement with the restriction of learning processes in the referenced device under low operating voltage. Contrarily, pulse‐interval‐dependent PPF index (Figure 4C) and pulse‐number‐dependent EPSC (Figure 4D) of SAMs‐based devices show that interfacial engineering enables and strengthens the learning processes in synaptic TFTs. Meanwhile, a higher PPF index usually suggests a lower rate of EPSC decay in synaptic devices, which is similar to the memory process in the human brain. As an example, time‐dependent EPSC (Figure 4E) and the corresponding EPSC rehearsal processes (Figure 4F) of different types of devices stimulated by 20 consecutive light pulses are recorded. Long‐sustained EPSC can be identified in SAMs‐based devices under *V_DS_* of −1 mV, showing that devices with interfacial engineering can effectively mimic the memory processes. Comparably, EPSC rapidly decays in the referenced device under *V_DS_* of −1 mV, suggesting an accelerated forgetting process in response to the external stimulation due to that carrier transport is severely hampered by scattering and non‐radiative recombination. The above results suggest that interfacial engineering allows PEA_2_SnI_4_ TFTs to work as photonic synapses with efficient learning and memory capabilities under operating voltage that is even lower than the excitatory postsynaptic potential to activate biological synapses (>15 mV), which is essential for their practical applications.

**Figure 5:**
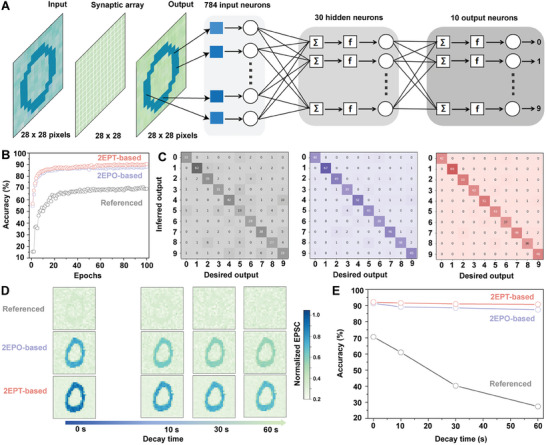
Image recognition based on PEA_2_SnI_4_ TFTs under *V_DS_* of −1 mV and ANNs. A) Schematic illustration of the neuromorphic visual system based on synaptic TFT arrays and ANNs for image preprocessing and recognition. B) Image validation set accuracy of MNIST patterns and C) the corresponding confusion matrix of training results after 100 epochs based on the referenced, 2EPO‐based, and 2EPT‐based devices. D) Evolution of output EPSC images as a function of EPSC decay time that mimics the memory processes. E) Image validation set accuracy of MNIST patterns as a function of EPSC decay time based on the referenced, 2EPO‐based, and 2EPT‐based devices.

Based on the synaptic TFT arrays (see details in the Figures S22 and S23, Supporting Information) that receive and store optical information and a three‐layer ANN, a neuromorphic visual system (**Figure** **5**A) mimicking the human visual system (Figure S24, Supporting Information) is constructed for supervised learning and validation of the Modified National Institute of Standards and Technology (MNIST) dataset. Specifically, synaptic TFT arrays are used as preprocessing hardware to record and memory input images, and the corresponding output EPSC mappings with different decay times (Figure S25, Supporting Information, including 0, 10, 30, and 60 s) are extracted for pattern recognition (handwritten digits ‘0′ – ‘9′) by ANNs. All TFT arrays are operated under *V_DS_* of −1 mV and are stimulated by 20 consecutive 405 nm light pulses of 12 µW cm^−2^, 100 ms, and 0.5 Hz. The three‐layer ANN is composed of 28 × 28 input neurons, 30 hidden neurons, and 10 output neurons and a weight‐updating algorithm of backpropagation (BP) is used.

Output EPSC images without decay (0 s) are first used for pattern recognition where synaptic TFT arrays serve as sensors accordingly. Recognition accuracy after 100 epochs based on the three types of devices from validation sets (Figure 5B) are shown to be 70.6% (referenced arrays), 91.4% (2EPO‐based arrays), 92.2% (2EPT‐based arrays). Meanwhile, the confusion matrix between input and output patterns after 10 (Figure S26A, Supporting Information), 30 (Figure S26B, Supporting Information), 50 (Figure S26C, Supporting Information), and 100 (Figure 5C) training epochs are provided. Such differences in recognition accuracy are in agreement with the enhanced EPSC magnitudes in interface‐engineered synaptic TFTs (Figure 4).

More importantly, output EPSC images with different decay times (10, 30, and 60 s) that mimic the memory ability in humans are then used for pattern recognition considering the long‐term memory ability of the interface‐engineered synaptic TFTs (Figure 4). Figure 5D shows a representative comparison of output images with different decay times of the three types of TFT arrays, and both SAMs‐based devices offer clear output images of the handwritten digit ‘0′ even after a 60 s decay while the memory ability of 2EPO‐based arrays was inferior to that of the 2EPT‐based arrays. For the referenced arrays, however, the output pattern becomes blurred after a forgetting time of 10 s, corresponding to the rapid decay of EPSC and restricted memory ability of the referenced device. Recognition accuracies based on output images with different decay times of the three types of arrays are shown in Figure 5E. Meanwhile, the confusion matrix between input and output patterns after 100 training epochs with different decay times (10, 30, and 60 s) are provided (Figure S27, Supporting Information). By extending the decay time to 60 s, recognition accuracy based on the referenced device is significantly reduced by 43.2%. Contrarily, recognition accuracies based on the 2EPO‐based and 2EPT‐based arrays are slightly decreased by 4% and 1.4%, respectively, showing that the interface‐engineered tin perovskite TFTs can effectively work as artificial photonic synapses for hardware toward neuromorphic visions under a low operating voltage of −1 mV. Such an operating voltage of hardware before ANN simulations is significantly lower than those of three‐terminal artificial photonic synapses based on conventional materials, 2D materials, organic materials, and perovskites by 1 to 3 magnitudes (Table S5, Supporting Information).

## 3 Conclusion

In this study, the role of dielectric/channel interface in determining the performance and functionality of perovskite TFTs is revealed. Specifically, interfacial engineering of PEA_2_SnI_4_ TFTs with SAMs is proposed to synergistically passivate defects on the surface of the SiO_2_ gate dielectric and defects in the sequentially deposited PEA_2_SnI_4_ channel, thereby suppressing the carrier scatterings and non‐radiative combinations during the transport of carriers. Two distinct benefits of interfacial engineering of tin perovskite TFTs are demonstrated. First, the interface‐engineered TFTs exhibit overall performance enhancement in carrier mobility, on/off current ratio, threshold voltage, dual‐scan hysteresis, subthreshold swing, and operational stability. Second, the optimization of the dielectric/channel interface enables efficient carrier transport under extremely low *V_DS_* down to −1 mV. Benefiting from the low‐voltage carrier transport characters, the interface‐engineered tin perovskite TFTs can efficiently work as photonic artificial synapses under an operating voltage of −1 mV, which is even lower than the excitatory postsynaptic potential to activate biological synapses (−15 mV). Consequently, a neuromorphic visual system based on the interface‐engineered TFT arrays operating at −1 mV is demonstrated for accurate pattern recognition, providing operational feasibility to the design of large‐scale‐integrated and wearable/implantable neuromorphic hardware.

## 4 Experimental Section

### Materials

Phenethylammoniumiodide (PEAI, 99.5%) was purchased from Xi'an Yuri Solar Co., Ltd. Tin(II) iodide (SnI_2_, 99.99%) was purchased from Advanced Election Technology Co., Ltd. 2‐(3,7‐dibromo‐10*H*‐phenothiazin‐10‐yl)ethyl) phosphonic acid (Br‐2EPT) and 2‐(3,7‐dibromo‐10*H*‐phenoxazine‐10‐yl)ethyl) phosphonic acid (Br‐2EPO) were purchased from Luminescence Technology Corp. N,N‐dimethylformamide (DMF, anhydrous, 99.8%) was purchased from Merck. Ethanol (EtOH, ≥99.5%) were purchased from Aladdin Reagent Ltd. Gold pellets (Au, 99.999%) were purchased from ZhongNuo Advanced Material Technology Co., Ltd. All the chemicals and solvents were used without further purification. The highly doped p‐type silicon wafers, with 100 nm of thermally oxidized SiO_2_, were obtained from Hefei Kejing Material Technology Co. Ltd.

### Device Fabrication

The perovskite solutions were prepared by dissolving PEAI (0.64 m, dissolved in DMF), SnI_2_ (0.32 m, dissolved in DMF)) in equal volume, and store it at 60 °C overnight in a N_2_‐filled glovebox with oxygen and water concentrations less than 1 ppm. Perovskite TFTs were fabricated on cleaned p^+^‐Si/SiO_2_ substrates using a bottom‐gate and bottom‐contact device configurations. The SiO_2_ dielectric layer has a thickness of 100 nm, with a capacitance per unit area of 34 nF cm^−2^. The substrates were cleaned using a standard cleaning procedure, including successive sonication in acetone, isopropanol, and deionized water for 15 min, respectively. After cleaning, the substrates were placed in a vacuum chamber for the deposition of 40 nm of Au as the source and drain electrodes. The channel length (L) and width (W) were 100 and 1000 µm, respectively. The substrates with electrodes were then treated with UV‐ozone for 30 min. Following the UV‐ozone treatment, the substrates were rapidly transferred inside the glove box for the deposition of perovskite films. To deposit SAMs interlayer, SAMs solutions (1 mg mL^−1^ in EtOH) were spun on SiO_2_ substrates at 4000 rpm for 30 s and annealed at 100 °C for 10 min. Hundred microliters DMF were added on the SAMs‐coated SiO_2_ substrate at 4000 rpm for 30 s to wash excess SAMs. The perovskite layers were spin‐coated at 4000 rpm for 50 s, followed by annealing at 100 °C for 10 min. Prior to the characterization of the TFTs, a portion of SiO_2_ was etched using a glass cutter to expose the gate electrode.

### Material Characterizations

XRD measurements were carried out with an X‐ray diffractometer (Rigaku Smartlab, Rigaku). The characteristic peak was measured by a 2D detector with Cu K‐α tube source (1.54184 Å) at the scanning speed of 10° min^−1^ and the step size of 0.01°. SEM measurements were carried out with a field‐emission scanning electron microscope (Crossbeam350, Carl Zeiss) with suitable magnification. AFM measurements were carried out with an atomic force microscope (MFP‐3D‐Infinity, Oxford Instruments Asylum Research). TRPL measurements were carried out with a fluorescence spectrometer (FLS1000, Edinburgh) and a 405‐nm laser. PL mappings were carried out with a laser scanning confocal microscope equipped with 532‐nm pulse laser and photon counting module. A band‐pass filter was used to collect PL with suitable wavelengths. XPS measurements were carried out using ULTRA‐PHI VersaProbe 4 (CoreTech Integrated Limited). XPS analysis was acquired using 100 W monochromated Al Kα (1486.6 eV) radiation and 100 µm X‐ray spot under 3.0 × 10^−7^ Pa.

### Device Characterizations

As for the TFTs device characterizations, the transfer curves, output curves, and bias stress stability tests were conducted using a semiconductor parameter analyzer (Keithley 4200) in a N_2_ filled box at room temperature. The measured mobility *μ* is in the saturation regime and the saturation mobility was calculated by $\mu =\left(2L/WC_{i}\right){\left(\partial \sqrt{I_{DS}}/\partial V_{GS}\right)}^{2}$ where *L*, *W*, and *C*_i_ are the channel length and width and dielectric areal capacitance, respectively. In this work, *L* = 100 µm, *W* = 1000 µm, and the SiO_2_ (100 nm) dielectric layer capacitance *C_i_* is 34 nF cm^−2^. The number of trapped carriers *∆N* was calculated using Δ*N*  = *C*Δ*V*/*q*  where *C* is the areal capacitance of the dielectric (34 nF cm^−2^) and *q* is the electron charge. Subthrehold swing *SS* are extracted from the transfer curves with *SS*  =  ∂*V_GS_*/∂(*logI_DS_*) where *V*_GS_ is the gate‐source voltage. Trap density (*N_T_*) can be calculated using the extracted *SS* values by *N_T_* =  [*q*(*SSq* × log *e*/*kT*) − 1](*C*/*q*) where *e* is the base of the natural logarithm, *k* is Boltzmann's constant, and *T* is the absolute temperature. A 405 nm laser diode was used as the light source for the optoelectronic characteristics of the device. The waveform generator converted the incident light into pulsed light. The light power intensity of incident irradiation was tuned and obtained by an optical power meter.

### Pattern Learning, Memory, and Recognition

First, a 7 × 7 array of tin perovskite synaptic TFTs was fabricated. Then, based on the synaptic post‐current data extracted from the 7 × 7 devices, the variances between devices were evaluated, considering metrics such as average and standard deviation. Subsequently, a numerical model of a neuromorphic array containing 28 × 28 pixels was simulated. Single‐layer neural network simulations based on photoelectric synaptic transistors were conducted using the MNIST handwritten dataset. Photocurrents were utilized at patterned locations, while dark currents were used in the background without patterns. Considering cross‐talk between adjacent pixels in the array, separate training sets containing 5000 images and validation sets containing 500 images were established. A test set comprising 500 images was generated from current measurements of devices at 0, 10, 30, and 60 s after illumination ended. The network consists of 784 input neurons (28 × 28) and 10 output neurons, with each neuron fully connected by a single synapse. Each synapse had its own synaptic weight (7840 weights), and each pixel of the input pattern (784 pixels) was fed to each input neuron. Subsequently, the input signal was scaled according to the synaptic weights, and then the scaled signal was fed into the output neurons for integration. Each output neuron uses a sigmoid function (y = (1 + exp(−x))^−1^) to generate a signal ranging from 0 to 1. Throughout all learning stages, backpropagation and weight updates were carried out based on computed errors.

## Conflict of Interest

The authors declare no conflict of interest.

## Author Contributions

Y.R., Y.D., and X.Z. contributed equally to this work. Y.C. and Q.S. conceived the idea. Y.C., T.W., and C.H. supervised the project. Y.R., D.Y., and T.W. prepared the devices and performed the material and device characterizations. X.Z. performed the simulations. All authors contributed to analyzing the data and commenting on the manuscript.

## Supporting information



Supporting Information

## Data Availability

The data that support the findings of this study are available from the corresponding author upon reasonable request.
