# Supplementary material for: Perovskite Thin‐Film Transistors for Ultra‐Low‐Voltage Neuromorphic Visions
Source: Adv Sci (Weinh). 2024 Nov 6;11(48):2410015. doi: 10.1002/advs.202410015 (PMC11672285; doi:10.1002/advs.202410015)
Supplement: Supplementary file 1 — Supporting Information [file ADVS-11-2410015-s001.pdf]

ADVANCED SCIENCE
Open Access

Supporting Information

for *Adv. Sci.*, DOI 10.1002/advs.202410015

Perovskite Thin-Film Transistors for Ultra-Low-Voltage Neuromorphic Visions

Yang Rong, De Yu, Xin Zhang, Tao Wang, Jie Wang, Yuheng Li, Tongpeng Zhao, Ruiqin He, Yuxin Gao, Can Huang*, Shumin Xiao, Jingkai Qin, Sai Bai, Huihui Zhu, Ao Liu, Yimu Chen* and Qinghai Song**

Supporting Information

of

Perovskite thin-film transistors for ultra-low-voltage neuromorphic visions

Yang Rong[1#], De Yu[1#], Xin Zhang[1#], Tao Wang[1]*, Jie Wang[1,2], Yuheng Li[3], Tongpeng Zhao[1], Ruiqin He[1], Yuxin Gao[1], Can Huang[1]*, Shumin Xiao[1], Jingkai Qin[4], Sai Bai[2], Huihui Zhu[5], Ao Liu[2], Yimu Chen[1]*, Qinghai Song[1]*

[1]Ministry of Industry and Information Technology Key Lab of Micro-Nano Optoelectronic Information System, Guangdong Provincial Key Laboratory of Semiconductor Optoelectronic Materials and Intelligent Photonic Systems, Harbin Institute of Technology (Shenzhen), Shenzhen, Guangdong, China, 518055.

[2]Institute of Fundamental and Frontier Sciences, University of Electronic Science and Technology of China, Chengdu, Sichuan, China, 611731.

[3]Sustainable Energy and Environment Thrust, The Hong Kong University of Science andTechnology (Guangzhou), Guangzhou, Guangdong, China, 511400.

[4]School of Integrated Circuits, Harbin Institute of Technology (Shenzhen), Shenzhen, Guangdong, China, 518055.

[5]School of Physics, University of Electronic Science and Technology of China, Chengdu, Sichuan, China, 611731.

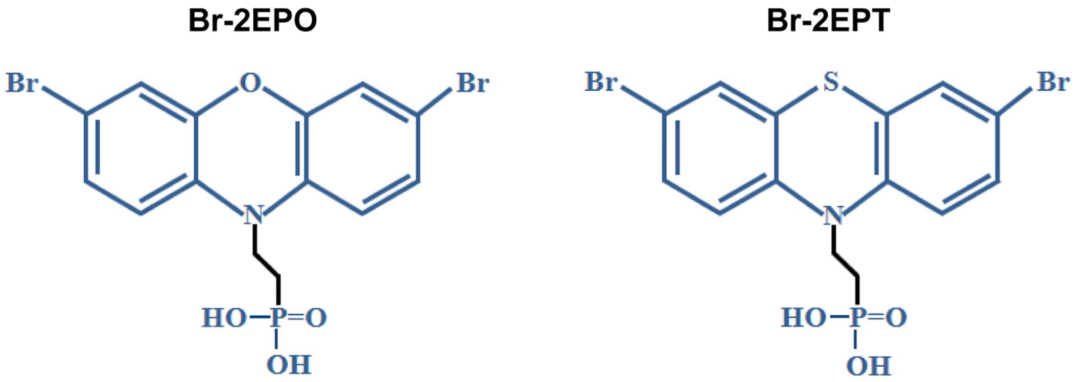

Figure S1. Chemical structures of Br-2EPO and Br-2EPT.

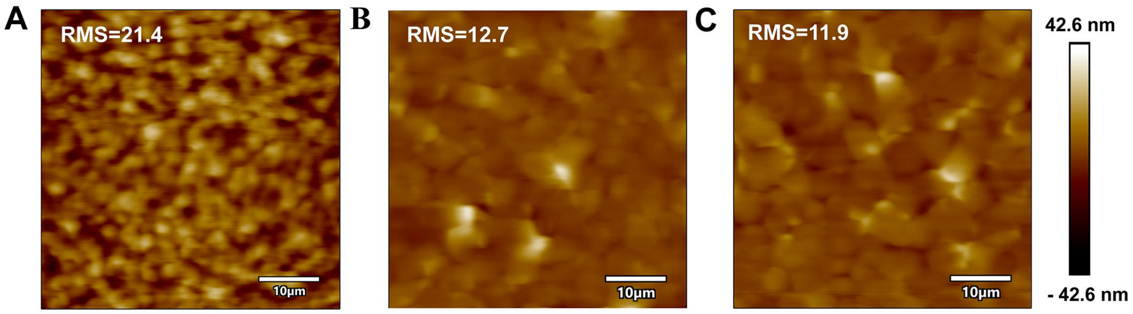

Figure S2. AFM images of (**A**) referenced, (**B**) 2EPO-based, and (**C**) 2EPT-based PEA_2SnI_4 thin films. Unit of roughness: nm.

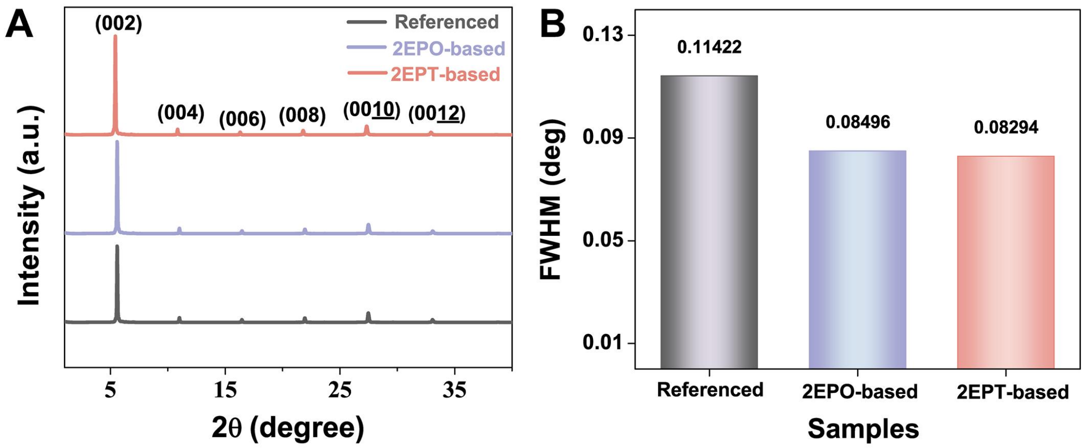

Figure S3. (**A**) XRD spectra and (**B**) FWHM of the (002) diffraction peak of the referenced, 2EPO-based, and 2EPT-based PEA_2SnI_4 thin films, respectively.

An increment of the diffraction peak intensity can be evident in both 2EPO-based and 2EPT-based PEA_2SnI_4 thin films. Besides, FWHM of the (002) peak in the referenced sample, and 2EPO-based and 2EPT-based PEA_2SnI_4 thin films are 0.114°, 0.085°, and 0.083°, respectively (Fig. S3B). The above phenomenon indicate an enhancement of the crystallinity in PEA_2SnI_4 thin films by interface engineering.

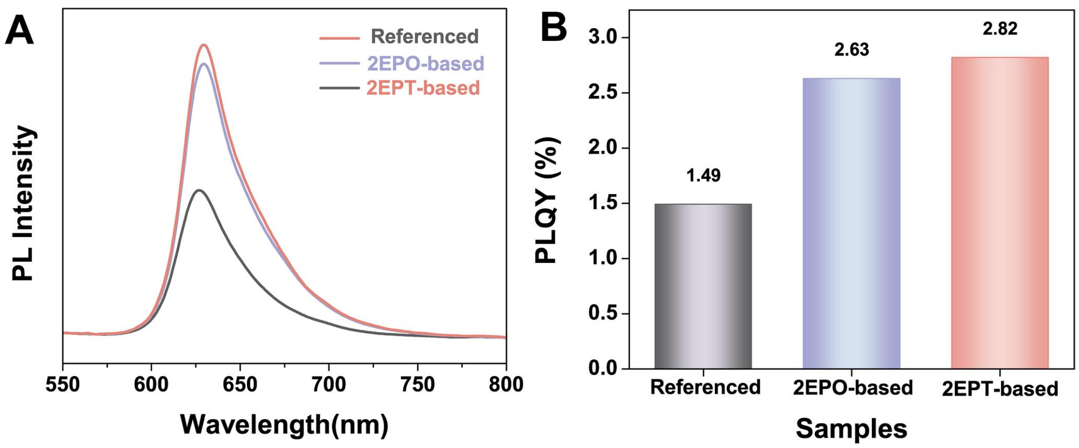

Figure S4. (**A**) Steady-state PL spectra and (**B**) PLQY of the referenced, 2EPO-based, and 2EPT-based PEA_2SnI_4 thin films.

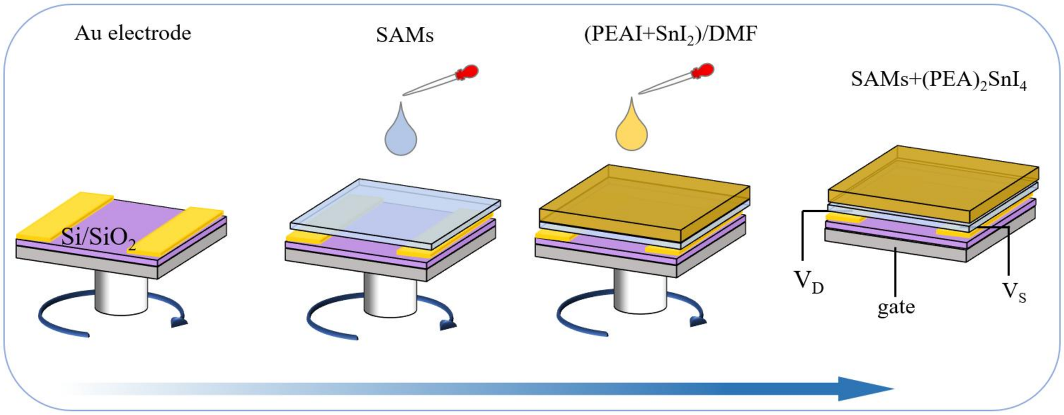

Figure S5. Schematic fabrication processes of PEA_2SnI_4 TFTs.

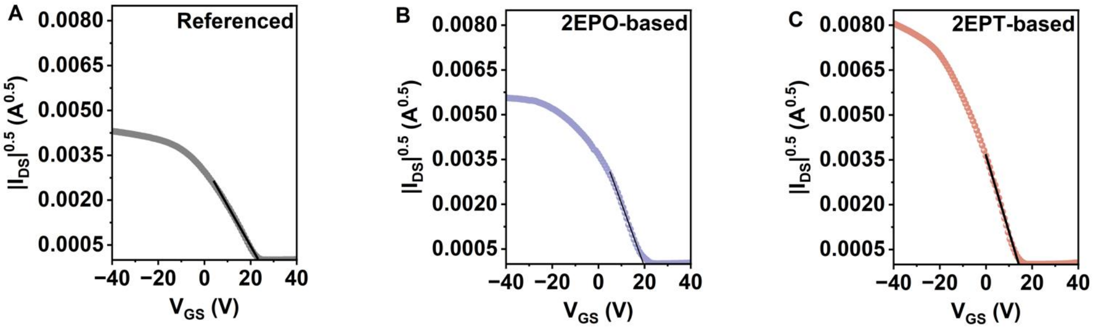

Figure S6. $|I_{DS}|^{0.5}$ of the perovskite TFTs based on the (A) referenced device, (B) 2EPO-devcie, and (C) 2EPT-device.

The extraction of threshold voltages is achieved by taking the square root of the transfer curves (Fig. S6). After that, the region where the drain current changes sharply with the change of gate voltage is selected and a linear fit is performed. The threshold voltage is then determined as the intercept of the tangent line with the horizontal axis.

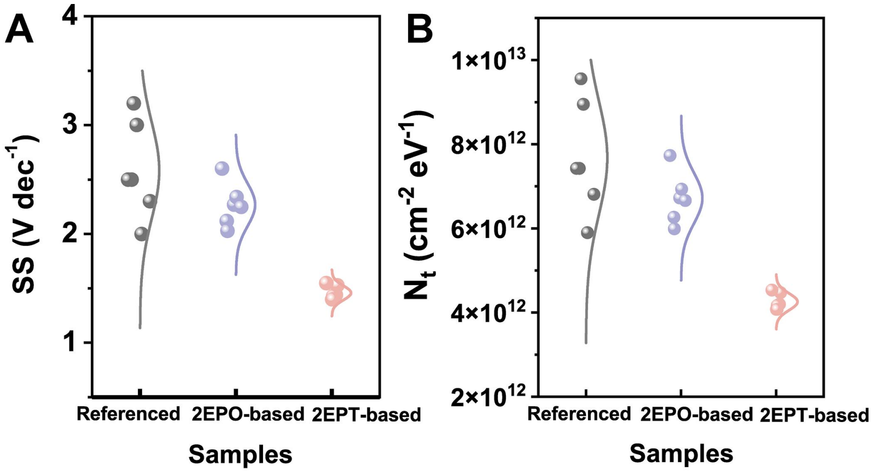

Figure S7. Statistical (**A**) *SS* and (**B**) N_t of the referenced, 2EPO-based, and 2EPT-based devices.

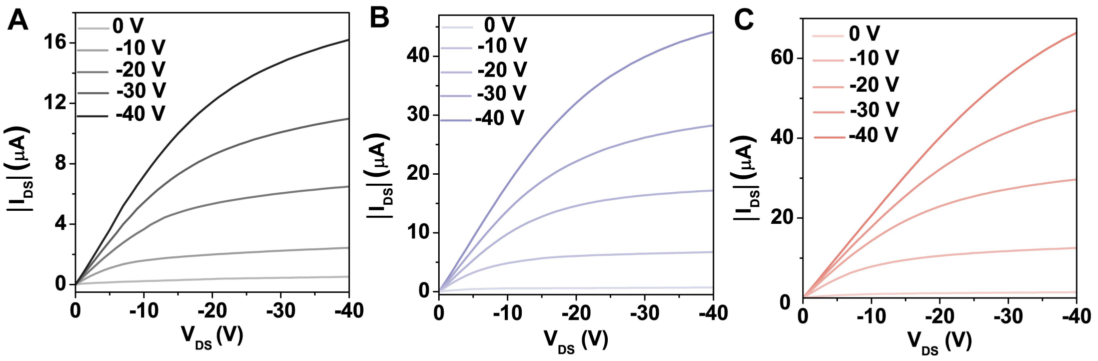

Figure S8. Typical output curves of (**A**) the referenced, **(B)** 2EPO-based, and **(C)** 2EPT-based devices.

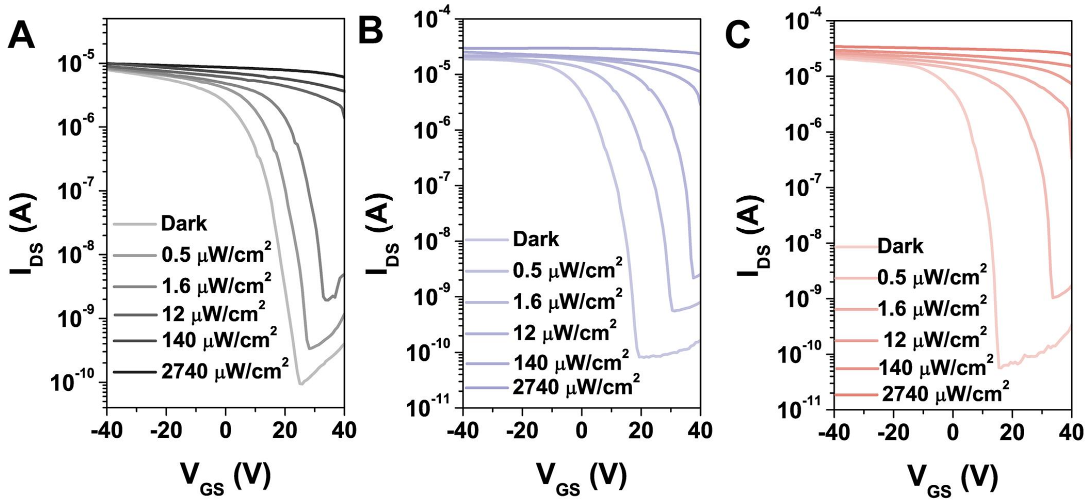

Figure S9. Transfer curves of (**A**) the referenced, (**B**) 2EPO-based, and (**C**) 2EPT-based devices under different illumination intensities.

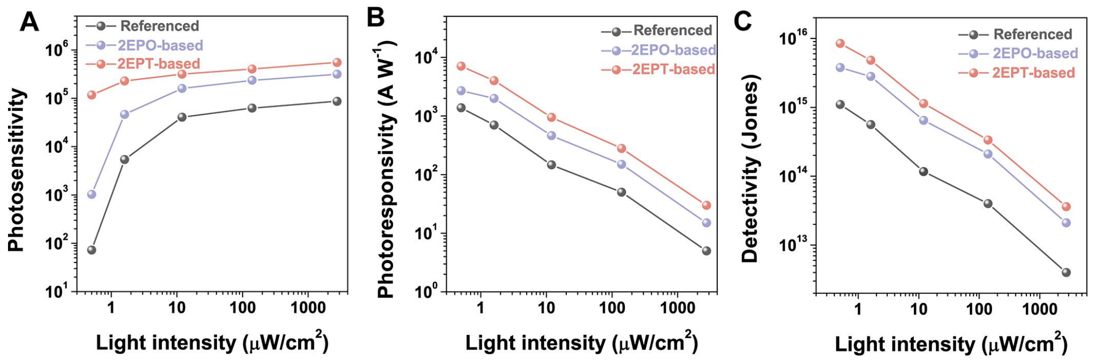

Figure S10. (**A**) Photosensitivity, (**B**) Photoresponsivity, and (**C**) Detectivity as functions of illumination intensity of the referenced, 2EPO-based, and 2EPT-based devices.

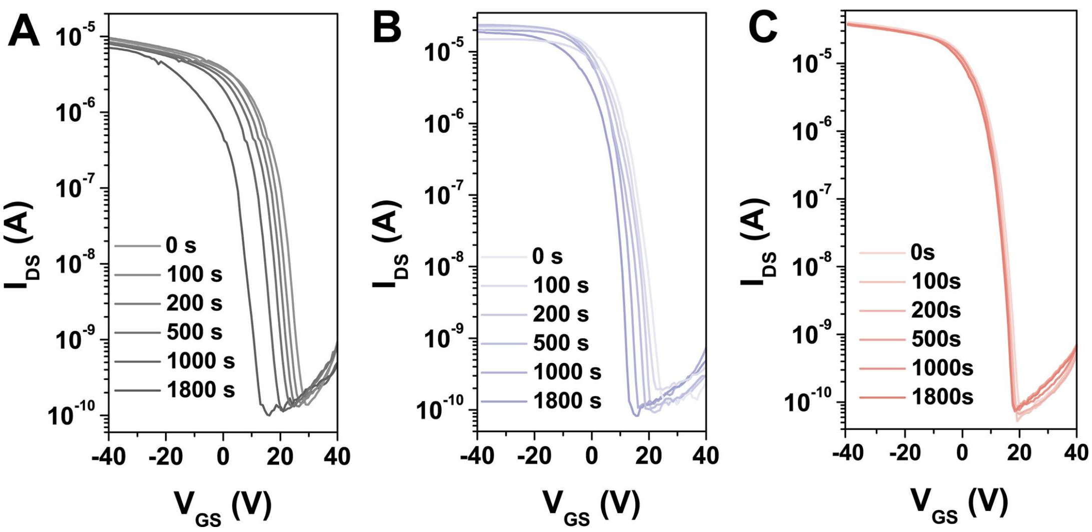

Figure S11. Bias-time-dependent transfer curves of (**A**) the referenced, (**B**) 2EPO-based, and (**C**) 2EPT-based devices during long-term bias stress measurements. All the devices are tested under a negative-bias condition (V_{GS} =-40 V, V_{DS} =-40 V) for different durations in a N_2-filled glovebox.

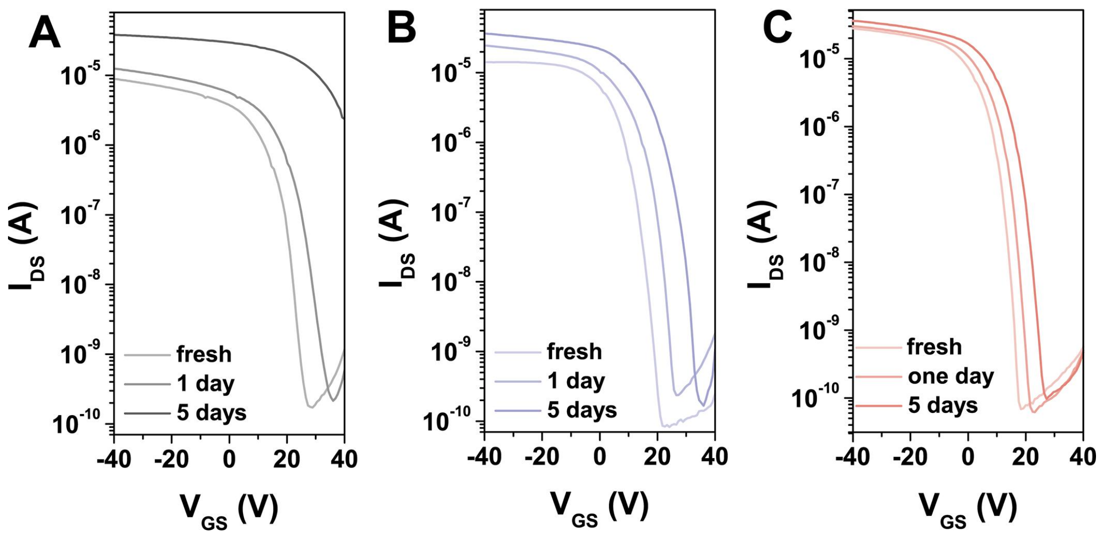

Figure S12. Transfer curves of (**A**) the referenced, (**B**) 2EPO-based, and (**C**) 2EPT-based devices during environmental stability test. All the devices are unencapsulated and tested in a N_2-filled glove box (oxygen level ~1 ppm; water level ~1 ppm).

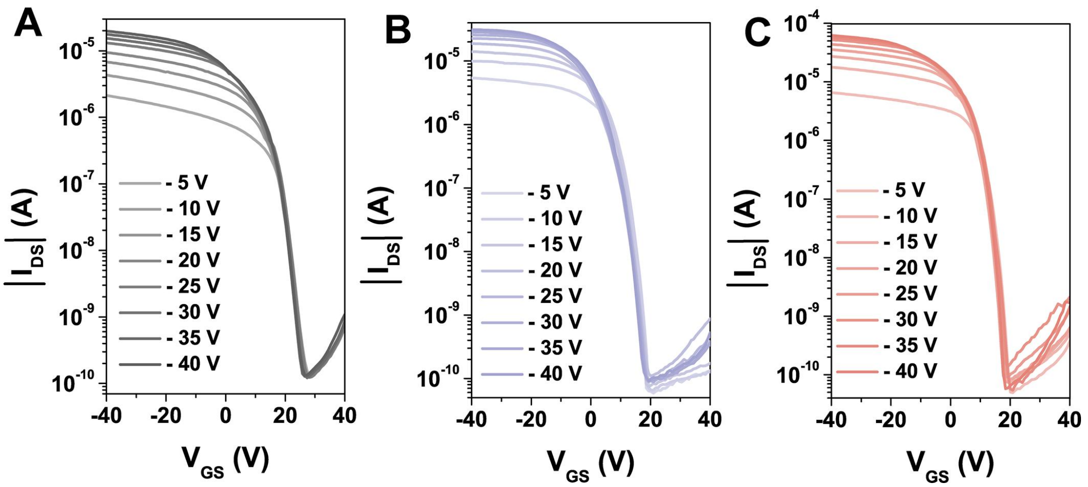

Figure S13. Transfer curves of (**A**) the referenced, (**B**) 2EPO-based, and (**C**) 2EPT-based devices under high V_{DS} ranging from -40 V to -5 V.

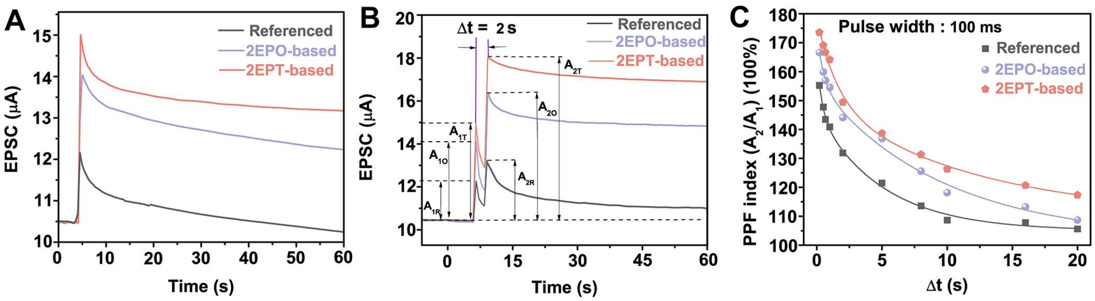

Figure S14. STP behaviors under V_{DS} of -20 V. (**A**) EPSC and (**B**) PPF behaviors of the three types of devices triggered by a single light pulse and a pair of light pulses, respectively. A_1 and A_2 represent the amplitude of the first and second EPSC, respectively. (**C**) Pulse-interval-dependent PPF index (A_2/A_1) of the three types of devices. Wavelength: 405 nm, pulse width: 100 ms, intensity: 12 μW cm^{-2}, V_{GS}: -20 V.

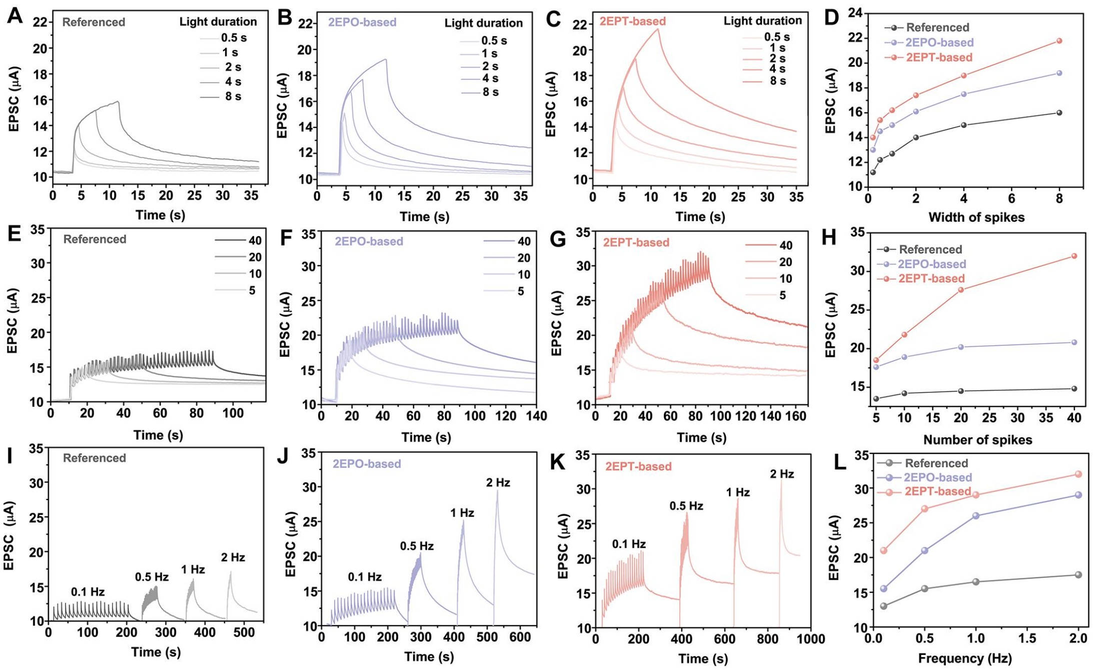

Figure S15. **EPSC behaviors controlled by different types of optical modulations under V_{DS} of -20 V.** (**A**-**C**) Pulse-duration-dependent EPSC and (**D**) the corresponding summary of the three types of devices. Wavelength: 405 nm, intensity: 12 μW cm^{-2}, V_{GS}: -20 V. (**E**-**G**) Pulse-number-dependent EPSC and (**H**) the corresponding summary of the three types of devices. Wavelength: 405 nm, pulse width: 100 ms, frequency: 0.5 Hz, intensity: 12 μW cm^{-2}, V_{GS}: -20 V. (**I**-**K**) Pulse-frequency-dependent EPSC and (**L**) the corresponding summary of the three types of devices. Wavelength: 405 nm, pulse width: 100 ms, intensity: 12 μW cm^{-2}, V_{GS}: -20 V.

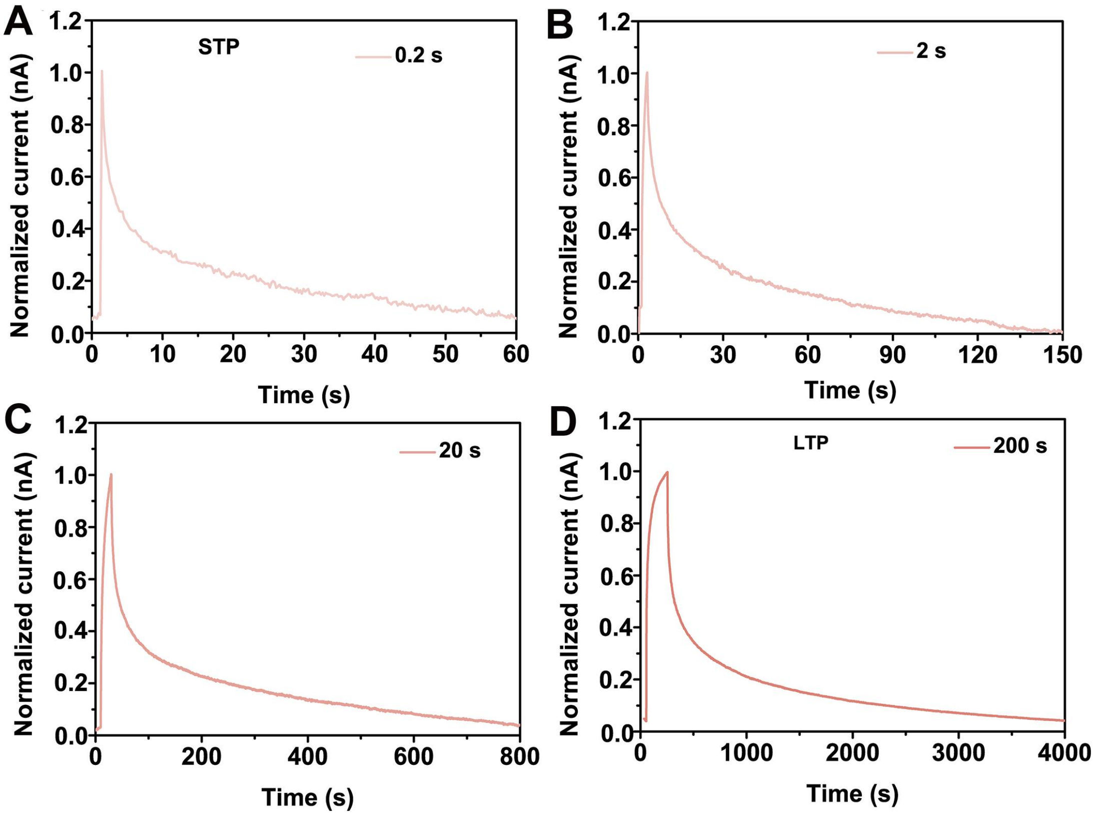

Figure S16. Transition from STP to LTP by extending light duration under V_{DS} of -20 V. EPSC behaviors of the 2EPT-based device after (**A**) 0.2 s, (**B**) 2 s, (**C**) 20 s, and (**D**) 200 s light illumination. Wavelength: 405 nm, intensity: 12 μW cm^{-2}, V_{GS}: -20 V.

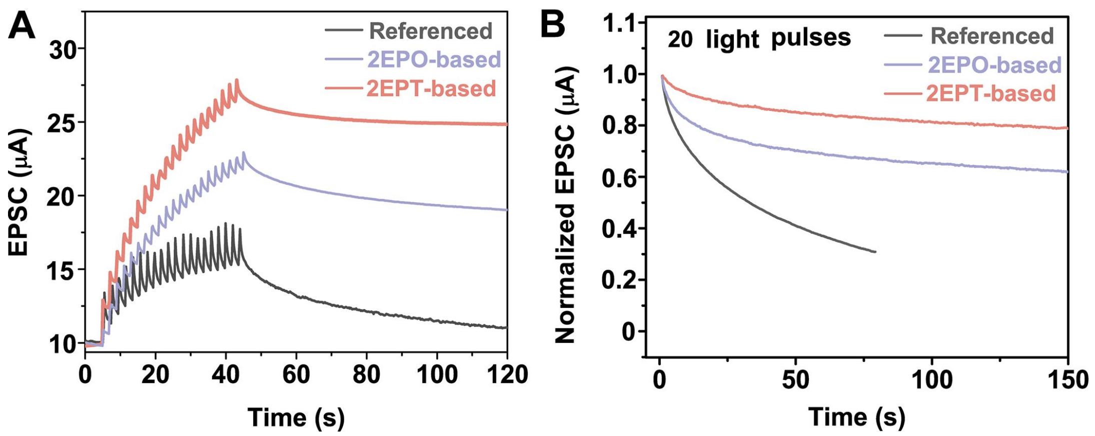

Figure S17. (**A**) EPSC behaviors and (**B**) the extracted EPSC decays of the three types of devices triggered by 20 consecutive light pulses. Wavelength: 405 nm, pulse width: 100 ms, frequency: 0.5 Hz, intensity: 12 μW cm^{-2}, V_{GS}: -20 V, V_{DS}: -20 V.

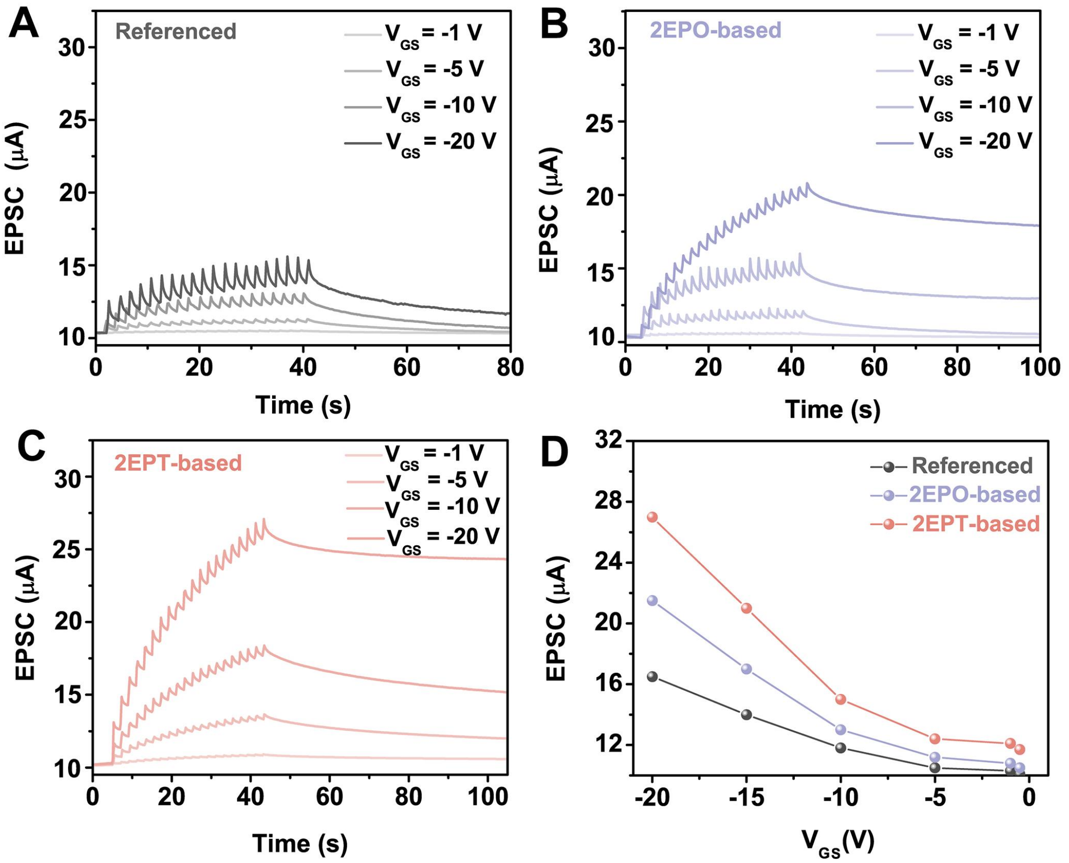

Figure S18. V_{GS}-dependent EPSC behaviors of (**A**) the referenced, (**B**) 2EPO-based, and (**C**) 2EPT-based devices triggered by 20 consecutive light pulses under V_{DS} of -20 V. (**D**) Summary of V_{GS}-dependent EPSC values of the three types of devices. Wavelength: 405 nm, pulse width: 100 ms, frequency: 0.5 Hz, intensity: 12 μW cm^{-2}.

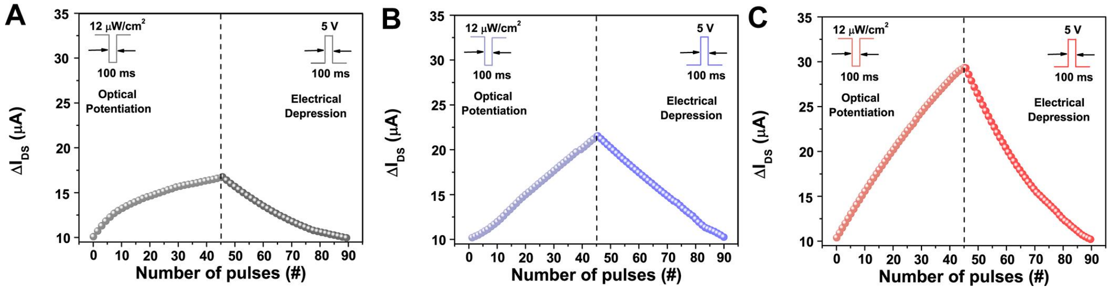

Figure S19. Optical-potentiation-electrical-depression operation based on the (A) referenced, (B) 2EPO-based devcie, and (C) 2EPT-based devcies, respectively.

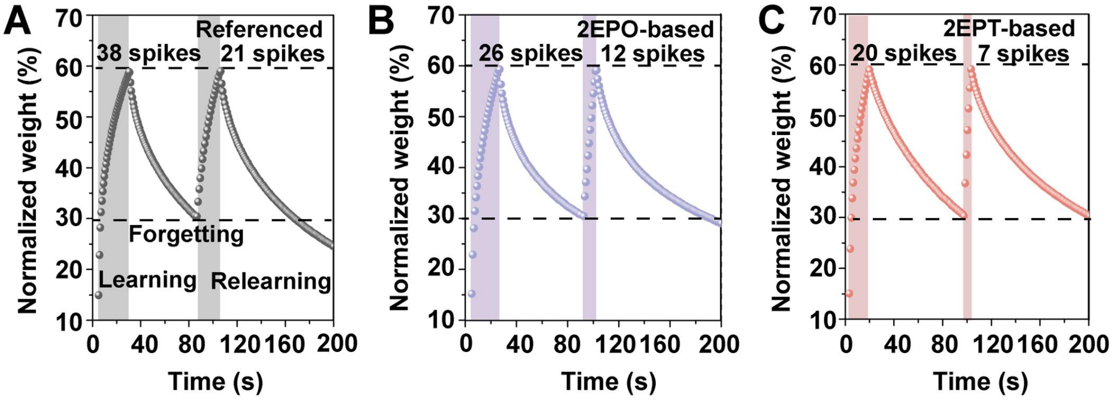

Figure S20. Learning-experience behavior (learning, forgetting, and relearning processes) of the (A) referenced, (B) 2EPO-based, and (C) 2EPT-based devcies.

To simulate the plasticity of the optoelectronic synapses for this learning experience behavior, continuous light pulses were employed to stimulate the device. As shown in Fig. S20, for the reference device, a synaptic weight level of 60% was attained with the assistance of 38 light pulses and gradually declined after the removal of the light pulses (the forgetting process). Continuous relearning through recall merely requires 21 light pulses to reach the previous cognitive level, indicating that the time needed for relearning is shorter than that for the initial learning. Under the same conditions, for the 2EPO-based device, 26 light pulses are necessary to reach 60% of the synaptic weight level, while relearning only demands 12 light pulses. Simultaneously, for the 2EPT-based device with superior optoelectronic performance, only 20 light pulses are needed to achieve the same synaptic weight, and relearning only requires 7 light pulses to reach the same cognitive level. Hence, the 2EPT-based device exhibits longer memory duration and stronger memory persistence, suggesting that it is more applicable in neural morphic visual system circuits.

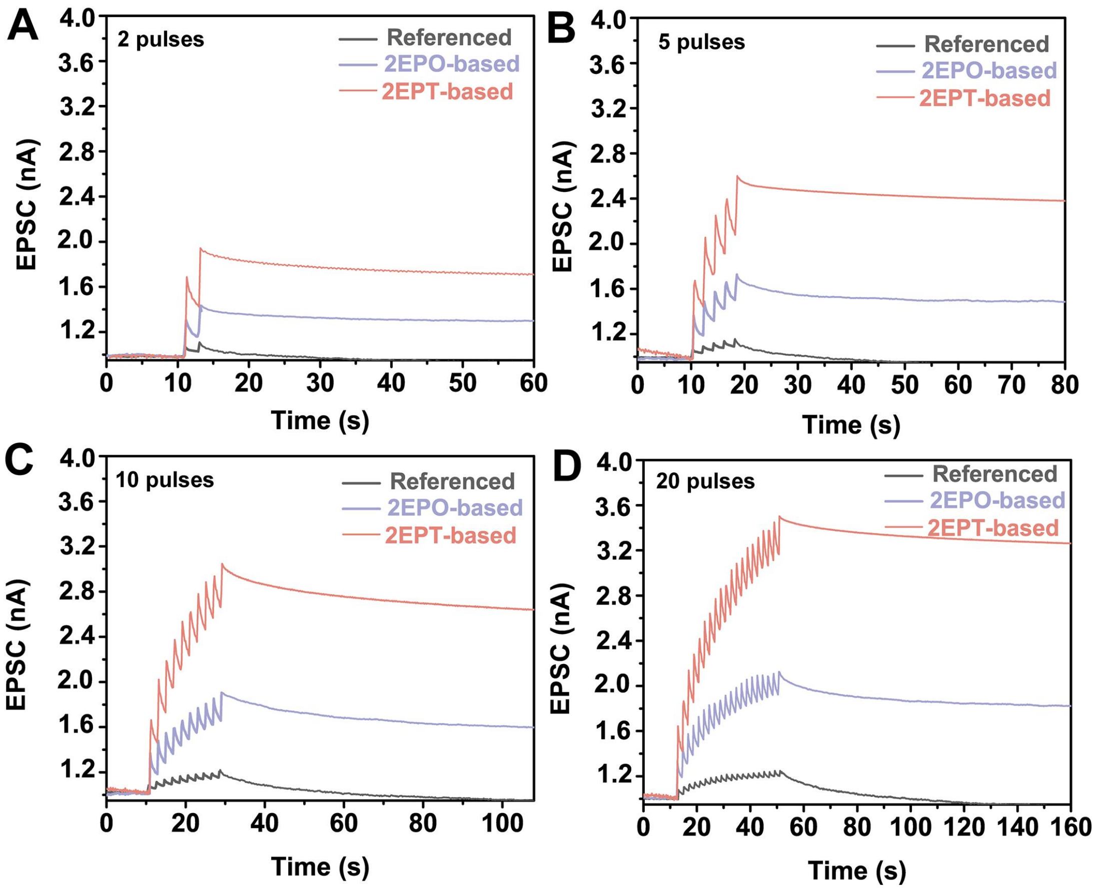

Figure S21. EPSC behaviors of the three types of devices under V_{DS} of -1 mV triggered by (**A**) 2, (**B**) 5, (**C**) 10, and (**D**) 20 consecutive light pulses. Wavelength: 405 nm, pulse width: 100 ms, frequency: 0.5 Hz, intensity: 12 μW cm^{-2}, V_{GS}: 0 V.

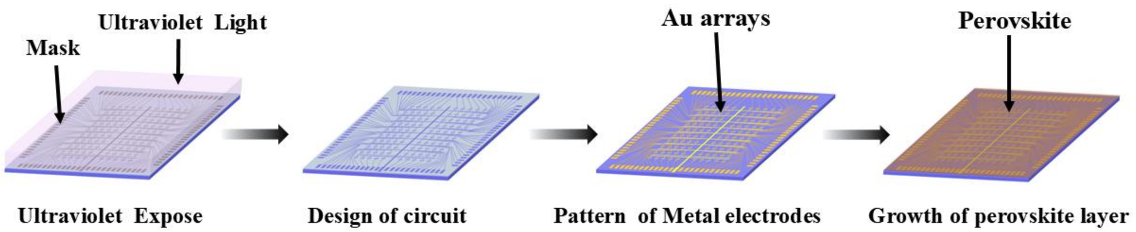

Figure S22. Schematic diagram of the fabrication process of the TFT arrays.

The schematic diagram of the fabrication process of the TFT arrays is depicted in Fig S22.

1) A Si/SiO_2 wafer is subjected to UV-ozone treatment for 15 minutes to improve its wettability. Subsequently, a thin layer of AZ nLOF 2020 negative photoresist was spin-coated onto the substrate at 4000 rpm for 60 s. Upon the completion of spin-coating, the substrate underwent annealing treatment at 110°C for 60 s.

2) The sample coated with the photoresist was exposed to UV light (365 nm) through a chrome/soda-line glass photomask on a Carl Suss MA6 mask aligner for 35 s, with an exposure dose of 55 mJ/cm^2. After exposure, the sample underwent annealing once again at 110°C for 65 s.

3) Upon the conclusion of annealing, the sample was immersed in AZ 300 MIF developer for 40 s to eliminate the unexposed photoresist, thereby exposing the microscale electrode areas. Subsequently, it was rinsed with water for 60 s to further purify the surface.

4) Cr and Au layers were deposited on the electrodes by means of electron beam physical vapor deposition (EB-PVD), with thicknesses of 5 nm and 35 nm, respectively. To eliminate the residual photoresist and isolate the electrodes, the silicon wafer was immersed in the AZ400T photoresist remover solution for more than 10 hours. This procedure guarantees that only the Cr/Au electrodes remain on the substrate, ready for the fabrication of the integrated array.

5) The perovskite layer was spin-coated at 4000 rpm for 50 s, followed by annealing at 100 °C for 10 min.

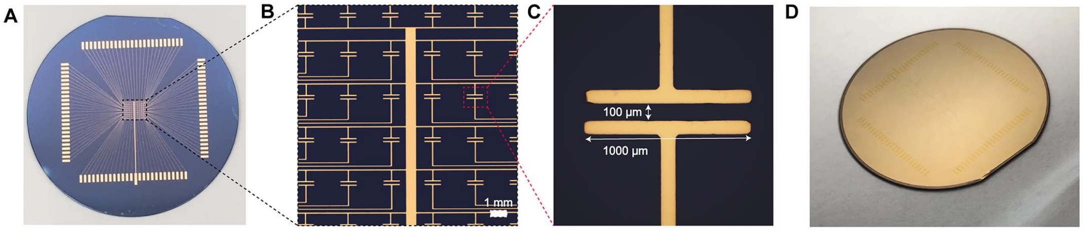

Figure S23. Synaptic TFT arrays demonstration. (**A-C**) Photographs of the designed circuits for synaptic TFT arrays. (**D**) Photograph of PEA_2SnI_4 synaptic TFT arrays.

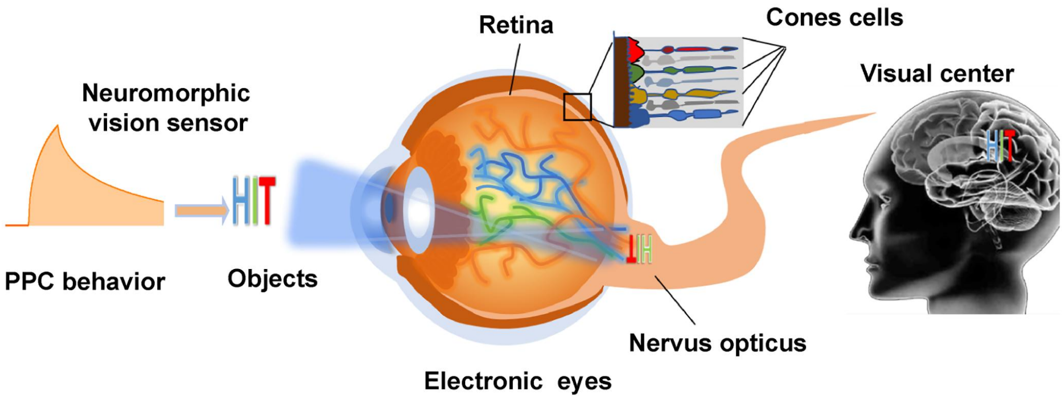

Figure S24. Schematic diagram of a human visual system. Visual information is initially detected and extracted through the retina in the human eye and further processed through the optic nerve in the visual cortex. In neuromorphic vision systems, a photonic synaptic device is used to simulate the perception and preprocessing functions of the human retina.

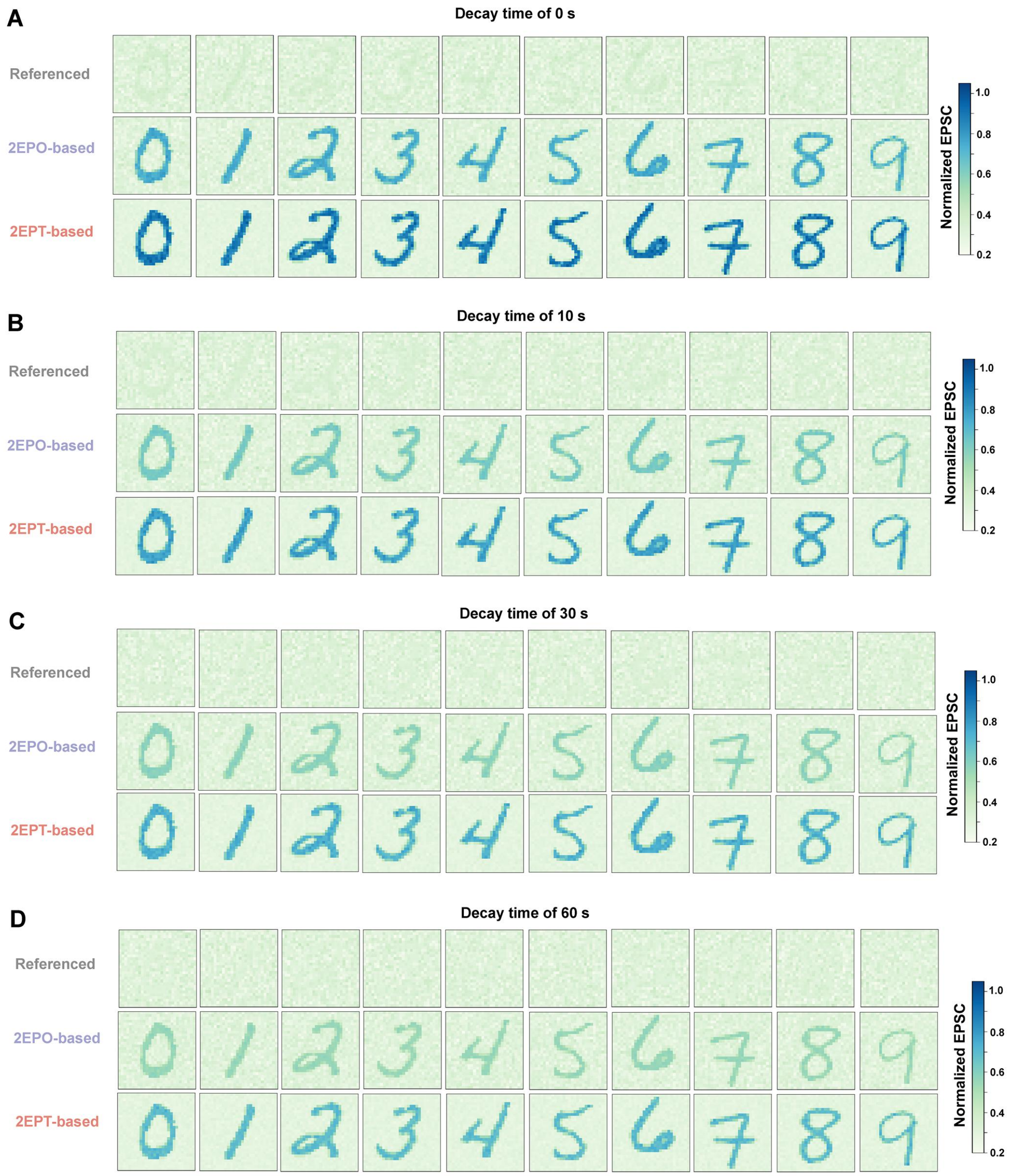

Figure S25. Evolution of output EPSC images as a function of EPSC decay time of (**A**) 0 s, (**B**) 10 s, (**C**) 30 s, and (**D**) 60 s by using the three types of devices. Such processes mimic the learning and memory processes in human.

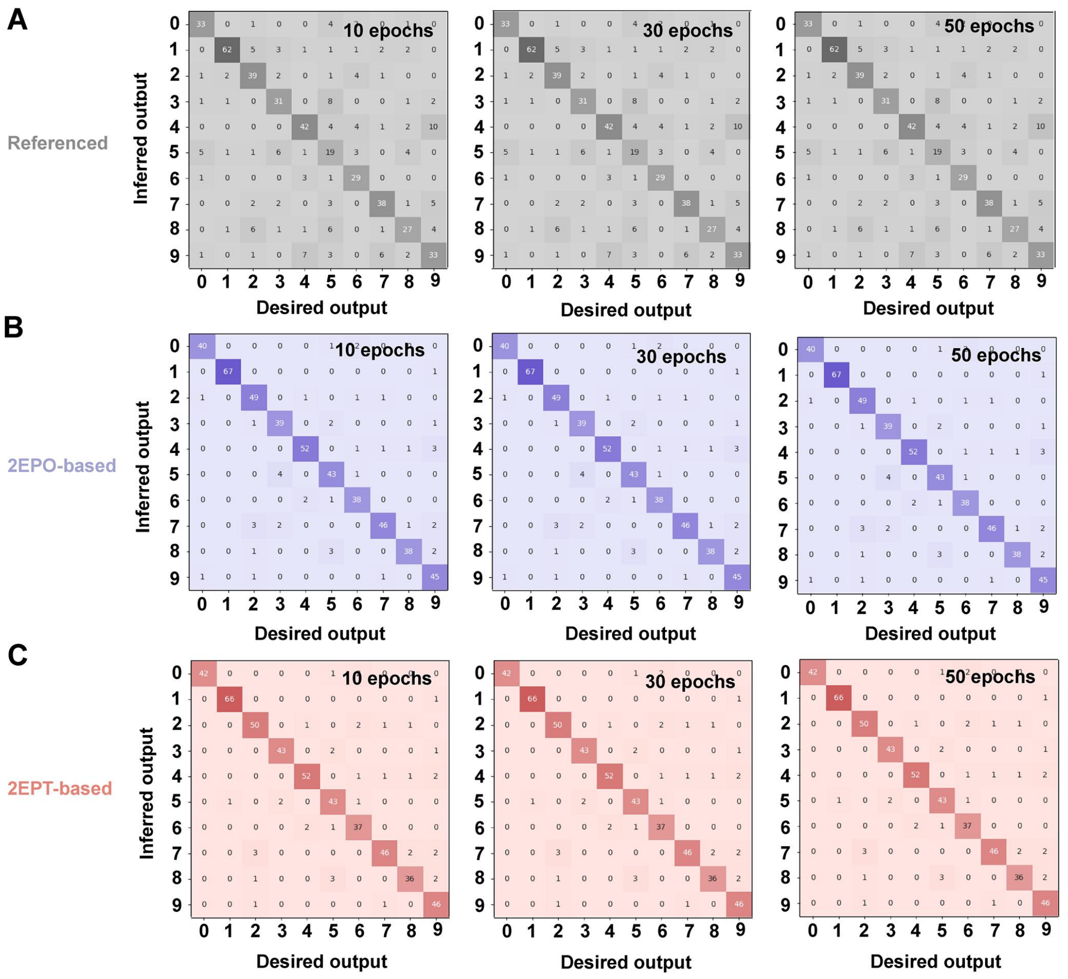

Figure S26. Confusion matrix of training results after 10, 30, and 50 epochs of the (**A**) referenced, (**B**) 2EPO-based, and (**C**) 2EPT-based devices by using output EPSC images with 0 s decay.

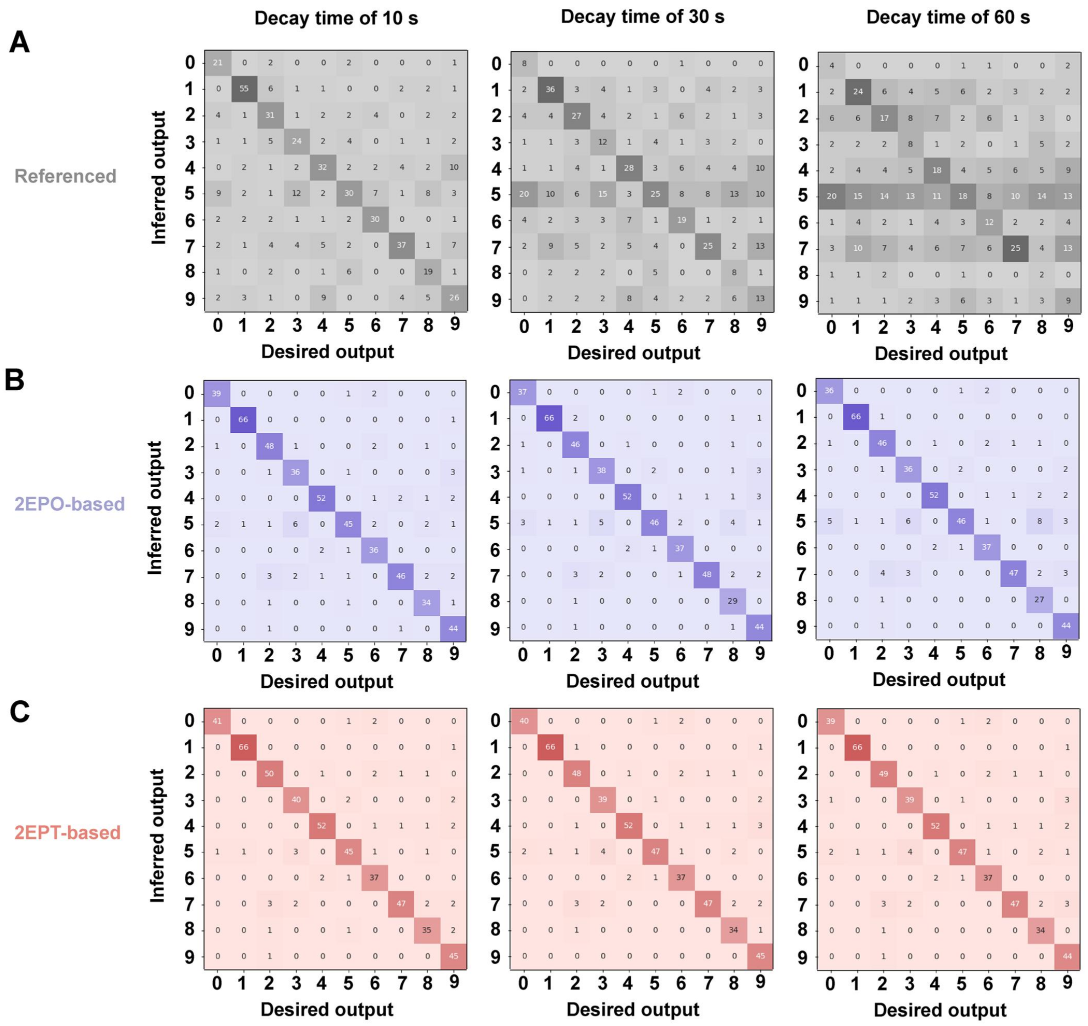

Figure S27. Confusion matrix of training results after 100 epochs of the (**A**) referenced, (**B**) 2EPO-based, and (**C**) 2EPT-based devices by using output EPSC images with different decay times (10, 30, and 60 s).

Table S1. Ratios of Si-O-Si, Si-O-H, and Si-O-P of the three types of SiO_2 substrates obtained from O 1*s* core level spectra.

	**Si-O-Si**	**Si-O-H**	**Si-O-P**
**UVO-treated**	51.8%	48.2%	-
**Br-2EPO-coated**	49.7%	27.4%	22.9%
**Br-2EPT-coated**	50.9%	26.4%	22.7%

Table S2. Ratios of Si-O-Si, Si-O-H, and Si-O-P of the three types of SiO_2 substrates obtained from Si 2*p* core level spectra.

	**Si-O-Si**	**Si-O-H**	**Si-O-P**
**UVO-treated**	63.2%	36.8%	-
**Br-2EPO-coated**	40.2%	26.3%	33.5%
**Br-2EPT-coated**	42.9%	23.0%	34.1%

Table S3: Summary of average lifetime of referenced, 2EPO-device and 2EPT-device.

	A_1	A_2	τ_1 (ns)	τ_2 (ns)	τ (ns)
**Referenced device**	495.06	34.99	0.87	3.12	1.32
**2EPO-device**	385.99	123.31	1.21	3.84	2.53
**2EPT-device**	327.80	137.96	1.59	4.98	3.51

The time resolved luminescence (TRPL) spectra were measured with an excitation of 405 nm and a detection wavelength of 629 nm, as shown in Fig. 2H. TRPL spectra were fitted using a biexponential equation as follows[1]:

$$I(t) = I_0 + A_1\, exp\left(-\frac{t}{\tau_1}\right) + A_2 exp\,(-\frac{t}{\tau_2})$$

where τ_1 and τ_2 were the short and long lifetimes, A_1 and A_2 were decay amplitudes of the component. The fitted short and long lifetimes are τ_1 =0.87 ns and τ_2 = 3.12 ns, τ_1=1.21 ns and τ_2=3.84 ns, and τ_1=1.59 ns and τ_2=4.98 ns for the referenced, 2EPO-based and 2EPT-based devcies, respectively (Table S3). The two decay channels are associated to two very different process namely surface component (fast) and bulk component (slow)[2]. After obtaining the above results, the average carrier lifetimes τ of each types of samples were calculated based on the following equation:

$$\tau = \frac{A_1 \times \tau_1^2 + A_2 \times \tau_2^2}{A_1 \times \tau_1 + A_2 \times \tau_2}$$

The average carrier lifetimes τ of the referenced, 2EPO-based, and 2EPT-based PEA_2SnI_4 thin films were respectively determined to be 3.12 ns, 3.84 ns, and 4.98 ns.

Table S4. Performance of the referenced, 2EPO-based, and 2EPT-based devices.

	**μ (cm^2 V^{-1} s^{-1})**	**On/off current ratio**	**V_{TH} (V)**	**SS (V dec^{-1})**
**Referenced**	0.148	1.02x10^5	22.56	2.5
**2EPO-based**	0.361	2.51x10^5	19.41	2.1
**2EPT-based**	0.57	6.56x10^5	15.53	1.4

Table S5. Summary of operational voltages and recognition accuracy of neuromorphic visual system based three-terminal synaptic arrays.

Device	Operational voltage	Pattern recognition accuracy	Ref.
**PS-b-P2VP/$MAPbBr_3$**	-1 V	90%	[3]
**$(PEA)_2PbI_4/HfO_2$**	0.1 V	99.8%	[4]
**PMMA/$CsPbBr_3$**	0.2 V	---	[5]
**Polymer electrolyte/$MAPbI_3$**	-0.5 V	81%	[6]
**PEA_2SnI_4/C8BTBT**	-1 V	92%	[7]
**$MAPbBr_3$ PDs/graphene**	0.5 V	44.1%	[8]
**BA_2PbBr_4/IZTO**	5 V	80%	[9]
**IGZO/$Cs_2AgBiBr_6$**	2 V	83.7%	[10]
**$CsBi_3I_{10}$/PMMA**	-5 V	92.1%	[11]
**SWCNT/$CsBi_3I_{10}$**	2 V	85.46%	[12]
**DPPDTT**	-0.6 V	97.19%	[13]
**h-BN/$ReSe_2$**	0.5 V	73%	[14]
**SnS_2/h-BN/CIPS**	0.1 V	93.63%	[15]
**NbS_2/MoS_2**	1 V	90%	[16]
**MoS_2/Ge**	-0.6 V	91.6%	[17]
**PZT/SiN_4/MoS_2**	50 mV	98.5%	[18]
**H_xWO_3/ZrO_2**	0.1 V	94%	[19]
**TiO_2/IGZO**	0.5 V	90.3%	[20]
**$SiO_2/Si_3N_4/SiO_2$**	1.5 V	91.8%	[21]
**Black phosphorus**	20 mV	96%	[22]
**Sb:In_2O_3**	50 mV	90%	[23]
**PEDOT:PSS/PEI**	10 mV	97%	[24]
**PEA_2SnI_4 TFTs**	-1 mV	92.2%	This work

Reference

1. Wang, T.; Hou, S.; Zhang, H.; Yang, Y.; Xu, W.; Ao, T.; Kang, M.; Pan, G.; Mao, Y., Highly controllable synthesis of $MAPbI_3$ perovskite nanocrystals with long carrier lifetimes and narrow band gap for application in photodetectors. *Journal of Alloys and Compounds* **2021,** *872*, 159589.

2. Shi, D.; Adinolfi, V.; Comin, R.; Yuan, M.; Alarousu, E.; Buin, A.; Chen, Y.; Hoogland, S.; Rothenberger, A.; Katsiev, K., Low trap-state density and long carrier diffusion in organolead trihalide perovskite single crystals. *Science* **2015,** *347* (6221), 519-522.

3. Lee, K.; Han, H.; Kim, Y.; Park, J.; Jang, S.; Lee, H.; Lee, S. W.; Kim, H.; Kim, Y.; Kim, T.; Kim, D.; Wang, G.; Park, C., Retina-Inspired Structurally Tunable Synaptic Perovskite Nanocones. *Advanced Functional Materials* **2021,** *31* (52), 2105596.

4. Hong, X.; Huang, Y.; Tian, Q.; Zhang, S.; Liu, C.; Wang, L.; Zhang, K.; Sun, J.; Liao, L.; Zou, X., Two-Dimensional Perovskite-Gated AlGaN/GaN High-Electron-Mobility-Transistor for Neuromorphic Vision Sensor. *Advanced Science* **2022,** *9* (27).

5. Wang, Y.; Lv, Z.; Chen, J.; Wang, Z.; Zhou, Y.; Zhou, L.; Chen, X.; Han, S. T., Photonic Synapses Based on Inorganic Perovskite Quantum Dots for Neuromorphic Computing. *Advanced Materials* **2018,** *30* (38).

6. Wei, H.; Yao, G.; Ni, Y.; Yang, L.; Liu, J.; Sun, L.; Zhang, X.; Yang, J.; Xiao, Y.; Zheng, F.; Xu, W., Flexible Electro-Optical Perovskite/Electrolyte Synaptic Transistor to Emulate Photoelectric-Synergistic Neural Learning Rules and Reflex-Arc Behavior. *Advanced Functional Materials* **2023,** *33* (46).

7. Xia, J.; Gao, C.; Peng, C.; Liu, Y.; Chen, P.-A.; Wei, H.; Jiang, L.; Liao, L.; Chen, H.; Hu, Y., Multidimensional Deep Ultraviolet (DUV) Synapses Based on Organic/Perovskite Semiconductor Heterojunction Transistors for Antispoofing Facial Recognition Systems. *Nano Letters* **2024**.

8. Pradhan, B.; Das, S.; Li, J.; Chowdhury, F.; Cherusseri, J.; Pandey, D.; Dev, D.; Krishnaprasad, A.; Barrios, E.; Towers, A., Ultrasensitive and ultrathin phototransistors and photonic synapses using perovskite quantum dots grown from graphene lattice. *Science advances* **2020,** *6* (7), eaay5225.

9. Park, Y.; Kim, M.-K.; Lee, J.-S., 2D layered metal-halide perovskite/oxide semiconductor-based broadband optoelectronic synaptic transistors with long-term visual memory. *Journal of Materials Chemistry C* **2021,** *9* (4), 1429-1436.

10. Huang, F.; Fang, F.; Zheng, Y.; You, Q.; Li, H.; Fang, S.; Cong, X.; Jiang, K.; Wang, Y.; Han, C.; Chen, W.; Shi, Y., Visible-light stimulated synaptic plasticity in amorphous indium-gallium-zinc oxide enabled by monocrystalline double perovskite for high-performance neuromorphic applications. *Nano Research* **2022,** *16* (1), 1304-1312.

11. Li, Y.; Wang, J.; Yang, Q.; Shen, G., Flexible Artificial Optoelectronic Synapse based on Lead-Free Metal Halide Nanocrystals for Neuromorphic Computing and Color Recognition. *Adv Sci (Weinh)* **2022,** *9* (22), e2202123.

12. Li, M.; Xiong, Z.; Shao, S.; Shao, L.; Han, S.-T.; Wang, H.; Zhao, J., Multimodal optoelectronic neuromorphic electronics based on lead-free perovskite-mixed carbon nanotubes. *Carbon* **2021,** *176*, 592-601.

13. Liu, X.; Dai, S.; Zhao, W.; Zhang, J.; Guo, Z.; Wu, Y.; Xu, Y.; Sun, T.; Li, L.; Guo, P.; Yang, J.; Hu, H.; Zhou, J.; Zhou, P.; Huang, J., All-Photolithography Fabrication of Ion-Gated Flexible Organic Transistor Array for Multimode Neuromorphic Computing. *Advanced Materials* **2024,** *36* (21).

14. Tsai, M.-Y.; Huang, C.-T.; Lin, C.-Y.; Lee, M.-P.; Yang, F.-S.; Li, M.; Chang, Y.-M.; Watanabe, K.; Taniguchi, T.; Ho, C.-H.; Wu, W.-W.; Yamamoto, M.; Wu, J.-L.; Chiu, P.-W.; Lin, Y.-F., A reconfigurable transistor and memory based on a two-dimensional heterostructure and photoinduced trapping. *Nature Electronics* **2023,** *6* (10), 755-764.

15. Wang, P.; Li, J.; Xue, W.; Ci, W.; Jiang, F.; Shi, L.; Zhou, F.; Zhou, P.; Xu, X., Integrated In-Memory Sensor and Computing of Artificial Vision Based on Full-vdW Optoelectronic Ferroelectric Field-Effect Transistor. *Advanced Science* **2023,** *11* (3).

16. Huang, P.-Y.; Jiang, B.-Y.; Chen, H.-J.; Xu, J.-Y.; Wang, K.; Zhu, C.-Y.; Hu, X.-Y.; Li, D.; Zhen, L.; Zhou, F.-C.; Qin, J.-K.; Xu, C.-Y., Neuro-inspired optical sensor array for high-accuracy static image recognition and dynamic trace extraction. *Nature Communications* **2023,** *14* (1).

17. Zhang, Y.; Wang, B.; Han, Z.; Shi, X.; Zhang, N.; Miao, T.; Lin, D.; Jiang, Z.; Liu, M.; Guo, H.; Zhang, J.; Hu, H.; Wang, L., Bidirectional Photoresponse in a Mixed-Dimensional MoS2/Ge Heterostructure and Its Optic-Neural Synaptic Behavior for Colored Pattern Recognition. *ACS Photonics* **2023,** *10* (5), 1575-1582.

18. Chen, J.; Zhu, Y.-Q.; Zhao, X.-C.; Wang, Z.-H.; Zhang, K.; Zhang, Z.; Sun, M.-Y.; Wang, S.; Zhang, Y.; Han, L.; Wu, X.; Ren, T.-L., PZT-Enabled MoS2 Floating Gate Transistors: Overcoming Boltzmann Tyranny and Achieving Ultralow Energy Consumption for High-Accuracy Neuromorphic Computing. *Nano Letters* **2023,** *23* (22), 10196-10204.

19. Cui, J.; An, F.; Qian, J.; Wu, Y.; Sloan, L. L.; Pidaparthy, S.; Zuo, J.-M.; Cao, Q., CMOS-compatible electrochemical synaptic transistor arrays for deep learning accelerators. *Nature Electronics* **2023,** *6* (4), 292-300.

20. Kim, J.; Song, S.; Lee, J. M.; Nam, S.; Kim, J.; Hwang, D. K.; Park, S. K.; Kim, Y. H., Metal-Oxide Heterojunction Optoelectronic Synapse and Multilevel Memory Devices Enabled by Broad Spectral Photocarrier Modulation. *Small* **2023,** *19* (35).

21. Kwon, D.; Woo, S. Y.; Lee, K.-H.; Hwang, J.; Kim, H.; Park, S.-H.; Shin, W.; Bae, J.-H.; Kim, J.-J.; Lee, J.-H. J. S. a., Reconfigurable neuromorphic computing block through integration of flash synapse arrays and super-steep neurons. *Science Advances* **2023,** *9* (29), eadg9123.

22. Ahmed, T.; Tahir, M.; Low, M. X.; Ren, Y.; Tawfik, S. A.; Mayes, E. L. H.; Kuriakose, S.; Nawaz, S.; Spencer, M. J. S.; Chen, H.; Bhaskaran, M.; Sriram, S.; Walia, S., Fully Light-Controlled Memory and Neuromorphic Computation in Layered Black Phosphorus. *Advanced Materials* **2020,** *33* (10).

23. Mazumder, A.; Nguyen, C. K.; Aung, T.; Low, M. X.; Rahman, M. A.; Russo, S. P.; Tawfik, S. A.; Wang, S.; Bullock, J.; Krishnamurthi, V.; Syed, N.; Ranjan, A.; Zavabeti, A.; Abidi, I. H.; Guo, X.; Li, Y.; Ahmed, T.; Daeneke, T.; Al-Hourani, A.; Balendhran, S.; Walia, S., Long Duration Persistent Photocurrent in 3 nm Thin Doped Indium Oxide for Integrated Light Sensing and In-Sensor Neuromorphic Computation. *Advanced Functional Materials* **2023,** *33* (36).

24. van de Burgt, Y.; Lubberman, E.; Fuller, E. J.; Keene, S. T.; Faria, G. C.; Agarwal, S.; Marinella, M. J.; Alec Talin, A.; Salleo, A., A non-volatile organic electrochemical device as a low-voltage artificial synapse for neuromorphic computing. *Nature Materials* **2017,** *16* (4), 414-418.
